# Elemental Depth Profiling of Intact Metal–Organic Framework Single Crystals by Scanning Nuclear Microprobe

**DOI:** 10.1021/jacs.1c08550

**Published:** 2021-11-02

**Authors:** Brian D. McCarthy, Timofey Liseev, Mauricio A. Sortica, Valentina Paneta, Wanja Gschwind, Gyula Nagy, Sascha Ott, Daniel Primetzhofer

**Affiliations:** † Department of Chemistry − Ångström Laboratory, Uppsala University, Box 523, 751 20 Uppsala, Sweden; ‡ Tandem Laboratory, Uppsala University, Box 529, 751 20 Uppsala, Sweden; § Department of Physics and Astronomy, Uppsala University, Box 516, 751 20 Uppsala, Sweden

## Abstract

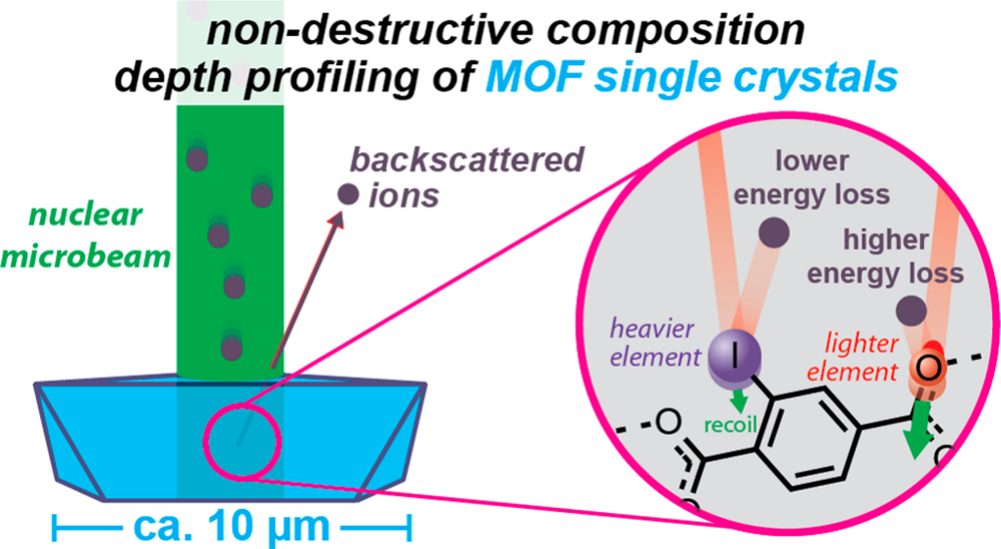

The growing field of MOF–catalyst composites often relies on postsynthetic modifications for the installation of active sites. In the resulting MOFs, the spatial distribution of the inserted catalysts has far-reaching ramifications for the performance of the system and thus needs to be precisely determined. Herein, we report the application of a scanning nuclear microprobe for accurate and nondestructive depth profiling of individual UiO-66 and UiO-67 (UiO = Universitetet i Oslo) single crystals. Initial optimization work using native UiO-66 crystals yielded a microbeam method which avoided beam damage, while subsequent analysis of Zr/Hf mixed-metal UiO-66 crystals demonstrated the potential of the method to obtain high-resolution depth profiles. The microbeam method was further used to analyze the depth distribution of postsynthetically introduced organic moieties, revealing either core–shell or uniform incorporation can be obtained depending on the size of the introduced molecule, as well as the number of carboxylate binding groups. Finally, the spatial distribution of platinum centers that were postsynthetically installed in the bpy binding pockets of UiO-67-bpy (bpy = 5,5′-dicarboxyy-2,2′-bipyridine) was analyzed by microbeam and contextualized. We expect that the method presented herein will be applicable for characterizing a wide variety of MOFs subjected to postsynthetic modifications and provide information crucial for their optimization as functional materials.

## Introduction

The fusion of classic *molecular* chemistry with *materials* chemistry holds enormous practical promise. Among others, the use of metal–organic frameworks (MOFs) as solid material scaffolds to host molecular catalytic units for the electrochemical conversion of energy-poor feedstocks like CO_2_ and water into energy-rich products is one such conceivable application.^1−15^ Incorporation of the catalytic sites into a MOF can be done either during solvothermal synthesis,^16^ postsynthetically by covalently modifying existing linkers using organic chemistry,^17^ by metalation of binding sites within the as-prepared MOF,^18^ or by exchanging existing linkers for functionalized ones.^19^ The latter approach, colloquially termed postsynthetic exchange (PSE) or solvent-assisted linker exchange (SALE),^20^ is often recognized as the mildest method, useful for incorporation of thermally sensitive functionalities.^21,22^ Metalation of existing binding pockets to produce catalytic sites—such as the binding of metal precursors to 2,2′-bipyridine-based linkers—has ample precedence in the literature, and the resulting materials have been demonstrated to be potent reusable catalytic materials.^23,24^ Last, the potential of multivariate MOFs—wherein an almost infinite number of discrete domains can be imagined inside a single MOF crystal—suggests the possibility for as yet unheard of cooperative effects.^25,26^

While powerful, these postsynthetic approaches have a clear *spatial* question: what is the final distribution of the introduced species throughout the MOF crystal? Perhaps as important, how does one *measure* this distribution? These questions are especially pressing given the inherent mass-transport limitations associated with heterogeneous catalysis: if substrate and/or charge carriers must physically diffuse within a MOF’s pores to reach an active site, what fraction of an individual MOF crystal is actually active?^10^ Indeed, to quantitatively benchmark different catalytic MOFs, knowledge of the distribution of the catalysts within the MOF is crucial.

Assessing the spatial profile of postsynthetic modifications within MOFs has some precedent, with *uniform distribution* of postsynthetic modification and *core–shell distribution* having been identified as the two limiting situations.^27−32^ The majority of reports for determining compositional depth profiles in MOFs rely on microspectroscopic (e.g., fluorescence or mapping Raman microspectroscopy) means of detection which require the growth of single crystals large enough (100–1000 μm) for visual or confocal analysis, and usually physical slicing of the crystals in order to obtain cross sections.^27,29,31,33−37^ While effective, these procedures may be time-consuming, destructive, and difficult for rapid multibatch analysis. A major limitation is that such large MOF crystals are often synthetically challenging to obtain and not suitable for catalytic applications where smaller crystals are likely necessary for quick intra-MOF diffusion of substrate.^10^ Consequently, there is a clear need for nondestructive elemental depth profiling of smaller—ca. 10 μm—MOF single crystals. Herein, we present such an analytical tool based on *elastic backscattering spectrometry* using a nuclear microprobe.

**Figure 1:**
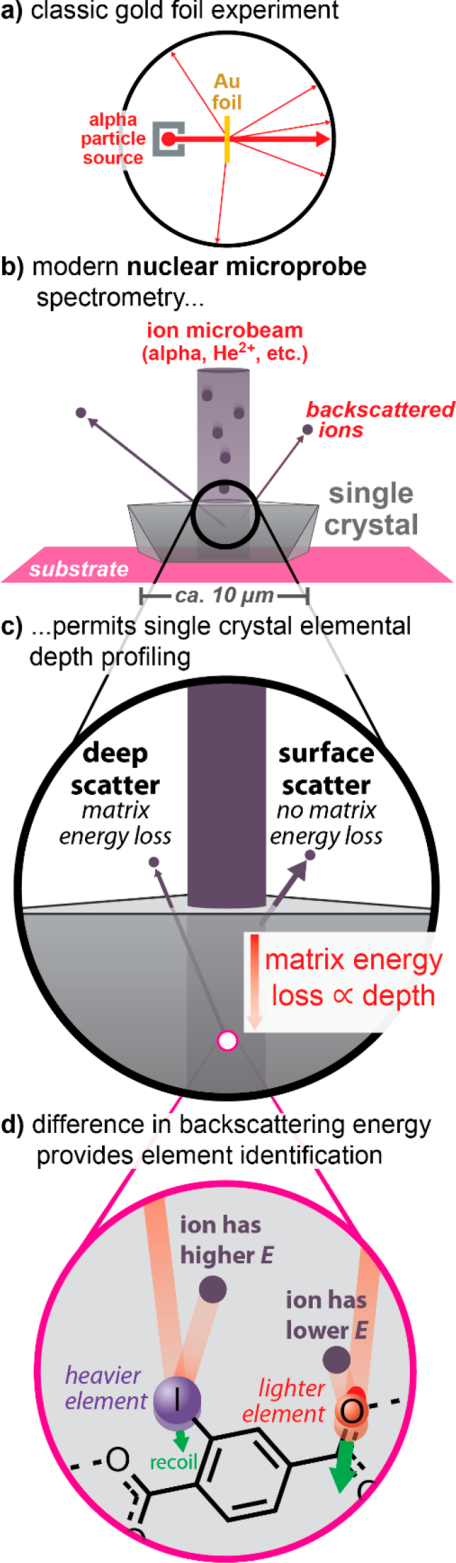
(a) Overview of the classic gold foil experiment of Geiger and Marsden, (b) overview of a modern nuclear microbeam as applied to elemental analysis of single crystals as discussed herein, and overview of how depth profiling with a nuclear microbeam is achieved using (c) knowledge of matrix energy loss (which is proportional to depth) combined with (d) the unique scattering kinematics associated with each element.

Modern ion scattering spectrometry is based on the classic gold foil experiments of Geiger and Marsden^38^ with analysis by Rutherford,^39^ wherein alpha particles were found to scatter at large angles off atomic nuclei (Figure 1a). In the form of highly accurate Rutherford backscattering spectrometry (RBS), the technique today enjoys applications in fields as diverse as solid–liquid interfaces^40^ and the noninvasive analysis of historical artifacts.^41−43^ In the past few years, we demonstrated, for the first time, that RBS could be used to determine the spatial distribution of linkers postsynthetically introduced into MOF particle ensembles.^28,44,45^

Herein, we show that, by using a nuclear *microbeam*, the methodology can be expanded from analysis of MOF aggregates to *single* crystals, thereby offering unprecedented precision in depth profiling using nondestructive backscattering spectrometry (Figure 1b).^45^ In this method, an ion beam with a diameter of at most a few micrometers first enters a crystal’s surface (Figure 1c). While passing through the crystal, the ions lose energy proportional to the penetration depth such that ions that scatter from deeper inside a sample lose more energy in the matrix (Figure 1c). Upon elastic scattering of the ion from an encountered nucleus, the ion loses additional energy, with trivial scattering kinematics providing a unique fingerprint of each element present in the sample (Figure 1d). Finally, as the ions travel back out of the sample (and ultimately to a detector), they lose further energy as a result of passing through the sample matrix. Thus, the element-specific scattering kinematics provides information on concentrations and the matrix energy loss provides depth perception; together, an elemental depth profile can be constructed.^46^ Furthermore, RBS is considered a method free from the need of external standards when establishing concentration ratios between constituents as the underlying physical principles have long been established.^46^

We demonstrate the utility of this method on UiO-type (UiO = Universitetet i Oslo) MOF single crystals in three case studies, where we assess the following: (1) the distribution of zirconium and hafnium in mixed-metal SBU UiO-66 obtained by solvothermal synthesis, (2) the distribution of linkers before and after postsynthetic introduction into UiO-66, using a heavy element iodine label as a spectrometric marker, and (3) spatial distribution of metal sites after postsynthetic metalation of UiO-67-bpy (bpy = [2,2′-bipyridine]-5,5′-dicarboxylic acid) with PtCl_2_. To our knowledge, the latter study is only the second of its kind that quantifies depth distribution of postsynthetic metalation.^26^

## Results and Discussion

### Avoiding Beam Damage

**Figure 2:**
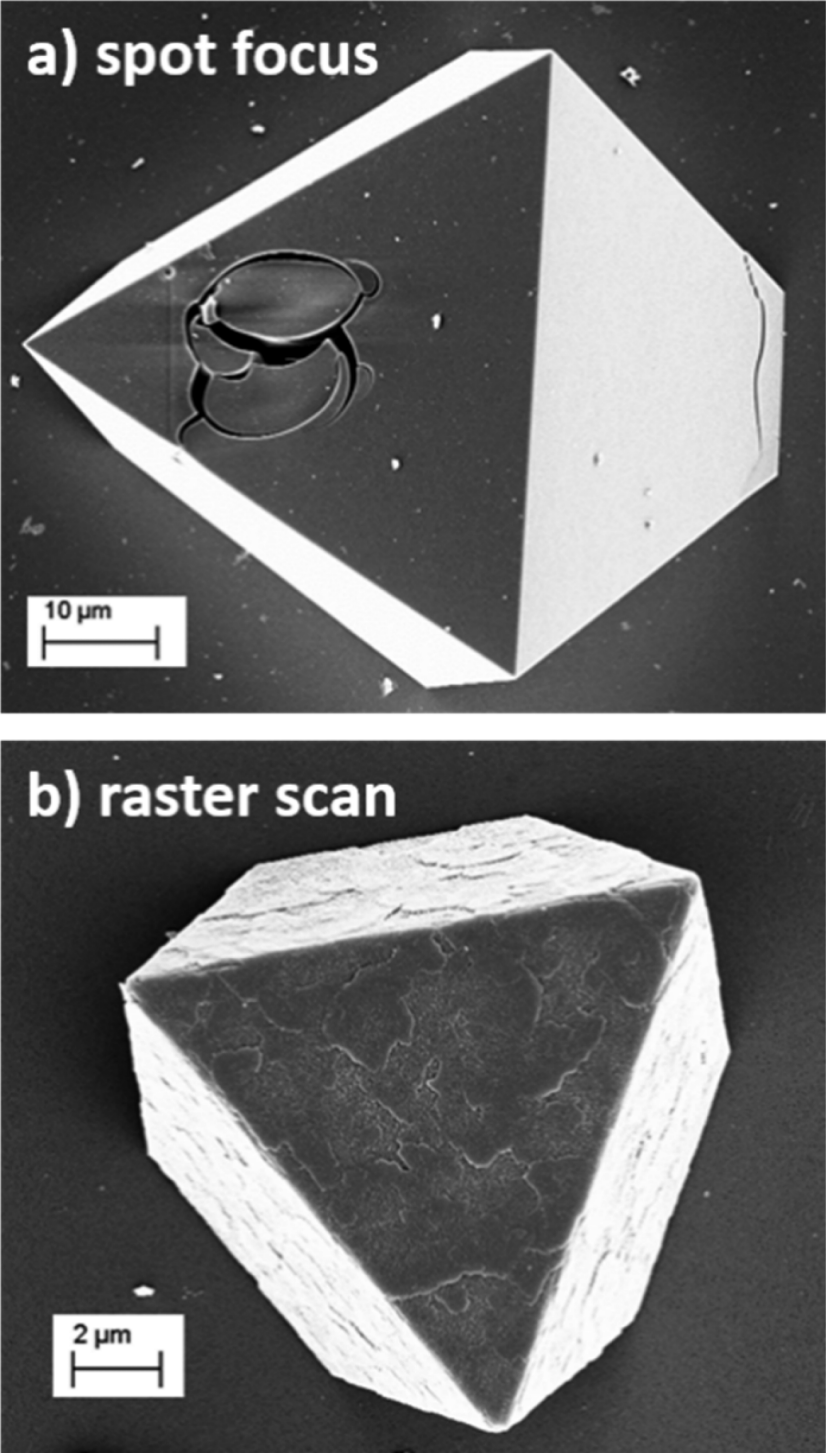
SEM images showing the effect of a 5 MeV He microbeam in different operational modes on UiO-66 single crystals on Si substrates for equivalent dose per unit area: (a) using a focused stationary beam; (b) using a raster scan beam.

Initial experiments with a microbeam using different projectile ions and beam energies ranging from 5 MeV He^+^ to 11 MeV C^3+^ were performed using the standard microbeam protocol of spot-focusing a 3–4 μm sized ion beam onto a single UiO-66 crystal grown on a Si wafer (see the Supporting Information and Figure S2 for details).^45^ Unfortunately, prolonged exposure of MOF crystals to focused ion bombardment caused permanent local damage to the crystals, as seen in SEM micrographs (Figure 2a) as well as in degradation of MOF-pertaining signals during the RBS measurement.

**Figure 3:**
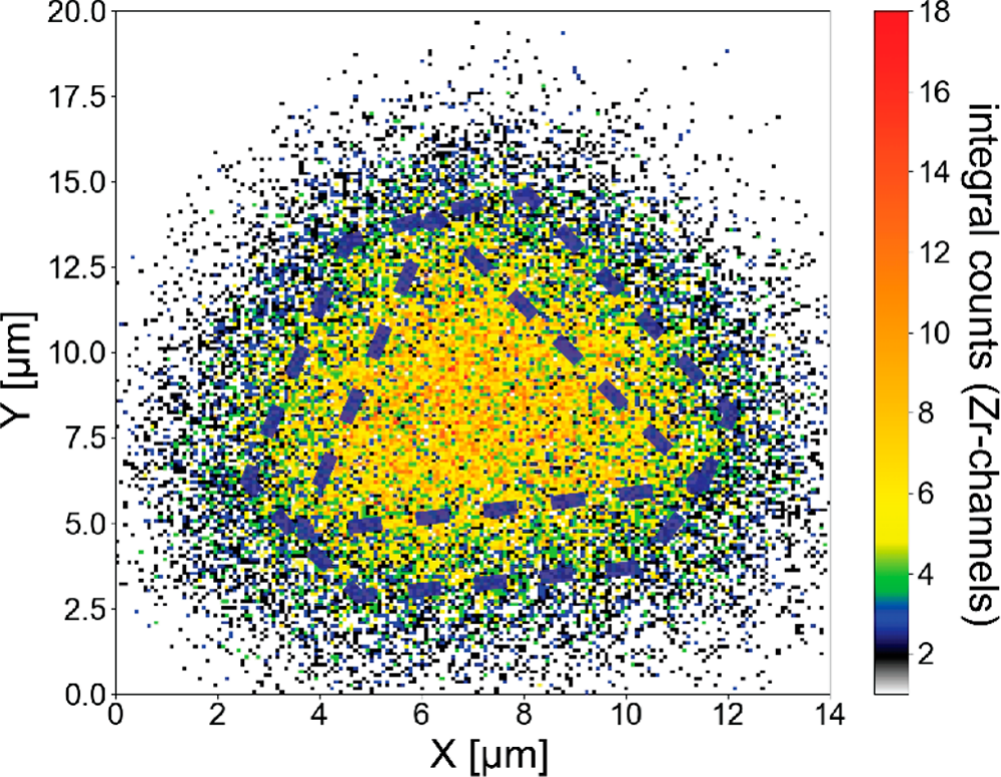
Example map of the signal corresponding to scattering of 5 MeV He^+^ primary ions from Zr for a single UiO-66 crystal. The dashed lines indicate the approximate location and orientation of the crystal.

To avoid this, a gentler approach was utilized wherein a beam of 5 MeV He^+^ was raster-scanned across an area which contained the crystal of interest, thereby allowing for more efficient thermal energy dissipation (Figure 2b). Plotting the integrated intensity of the Zr signal, which is well separable from the substrate signal, as a function of beam position, we can create elemental maps of the crystals (Figure 3). From these maps, depth profiles can be extracted by selecting the central region of the crystal in the map and summing up the individual energy spectra. Note that, while we selected a beam size of 3–4 μm, smaller beam sizes could be employed, though at the cost of increased measurement time due to the resulting decrease in current.

### Spatial Distribution of Hf in Mixed-Metal UiO-66 Single Crystals

With a nondamaging method in hand, a microbeam was used to analyze UiO-66 crystals which had been grown solvothermally on Si slides using a mixture of 70% zirconium oxychloride and 30% hafnium oxychloride (see the Supporting Information for details and Figure S3 for the XRD pattern). This MOF was chosen for a first proof-of-concept study, since the energy edge for Hf is well separated from that of Zr. Furthermore, mixed-metal SBUs (secondary building units) are well-known, with reported applications ranging from catalysis to gas sorption.^47^ As noted by Leus and co-workers, these mixed-metal MOFs (MM-MOFs) should be carefully studied to ensure that both metals are actually present in the same crystal, rather than a mix of pure metal MOFs—a fact easily missed if only conventional bulk analysis techniques like inductively coupled plasma (ICP) mass spectrometry are used.^47^

**Figure 4:**
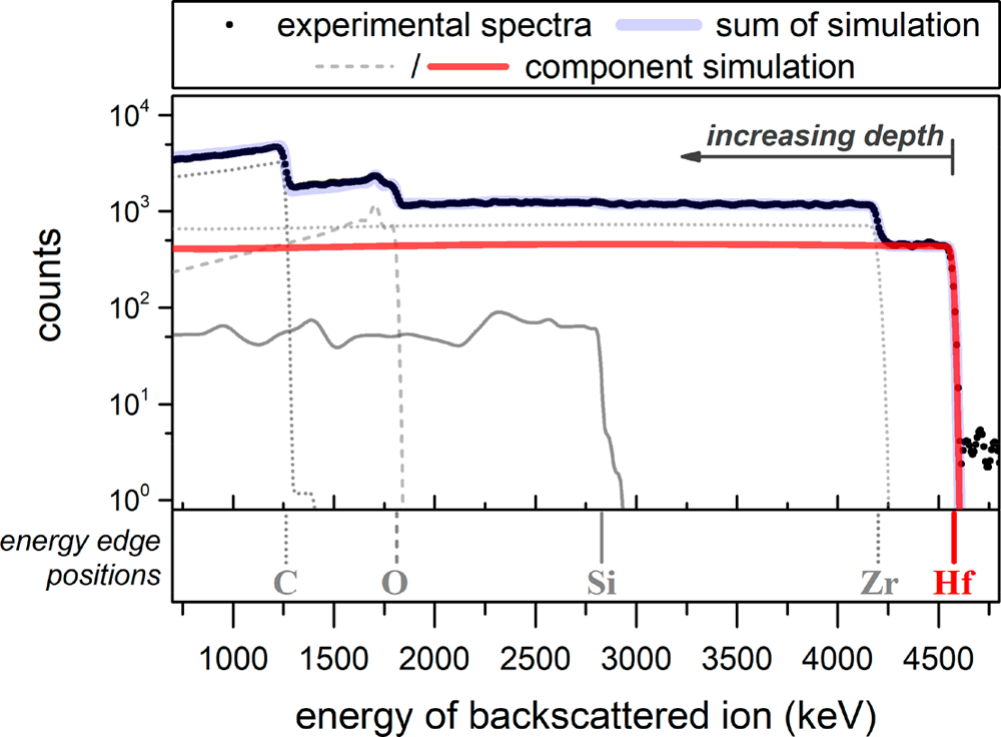
Experimental energy spectra and simulation fits of 5 MeV primary He ions backscattered from a mixed Zr/Hf UiO-66 single crystal on a Si wafer.

We hypothesized that Hf should be uniformly distributed in the resulting UiO-66 crystals given its chemical similarity to Zr,^48^ though core–shell architectures have been reported for MM-MOFs where the metals had nucleation rates differing by almost a factor of 100.^49^ Analysis by a raster-scanned nuclear ion beam of a single crystal revealed the presence of both Zr and Hf (Figure 4). For each element, the energy scale corresponds to its respective depth distribution, with the high-energy “edge” of the signal originating from the element at the surface of the crystal. Fitting of the data was performed by building a multilayer model which included the observed elements using SIMNRA simulating the expected RBS spectra.^50^ Both the simulation sum and individual element simulations are shown in Figure 4; the ratio of Zr and Hf was found to be 5:1. If the crystal composition matched exactly the composition of the initial solvothermal solution, a Zr to Hf ratio of ca. 2.3:1 would have been expected. The observed enhancement of Zr indicates that the crystallization kinetics favors Zr in the SBUs.

As seen in Figure 4, the Hf signal continues into the Zr signal, confirming that Hf was not present only on the crystal surface. Fitting of the data confirmed the ratio to stay constant within <10% over the whole probed depth of ca. 10 μm. This observation supports that a uniform distribution is expected for MOFs made of chemically similar metals. Further work will be necessary to probe if the SBUs themselves are of homogeneous composition, or if certain SBU stoichiometries are preferred, as found for mixed Zr/Ce UiO-66 MOFs.^51^

### Spatial Distribution of PSE

With results demonstrating that metal distributions in mixed-metal MOFs can be analyzed by a microbeam of single MOF crystals, we turned to analysis of MOFs which underwent postsynthetic exchange of linkers and/or modulator. Pre-evacuated UiO-66 single crystals of ca. 10 μm size on Si wafers were separately incubated in 250 mM methanolic solutions of either 3-iodobenzoic acid (iba), the monoester 3-iodo-4-(methoxycarbonyl)benzoic acid (monoester), or iodoterephthalic acid (ita) at 50 °C for 24 h, yielding UiO-66-iba, UiO-66-monoester, and UiO-66-ita, respectively. After the PSE process, the MOF@Si slides were washed in ethanol over 3 days, exchanging the solvent at least six times, followed by drying *in vacuo* before ion beam analysis. XRD confirmed retention of the UiO-66 crystal structure (Figures S4 and S5).

**Figure 5:**
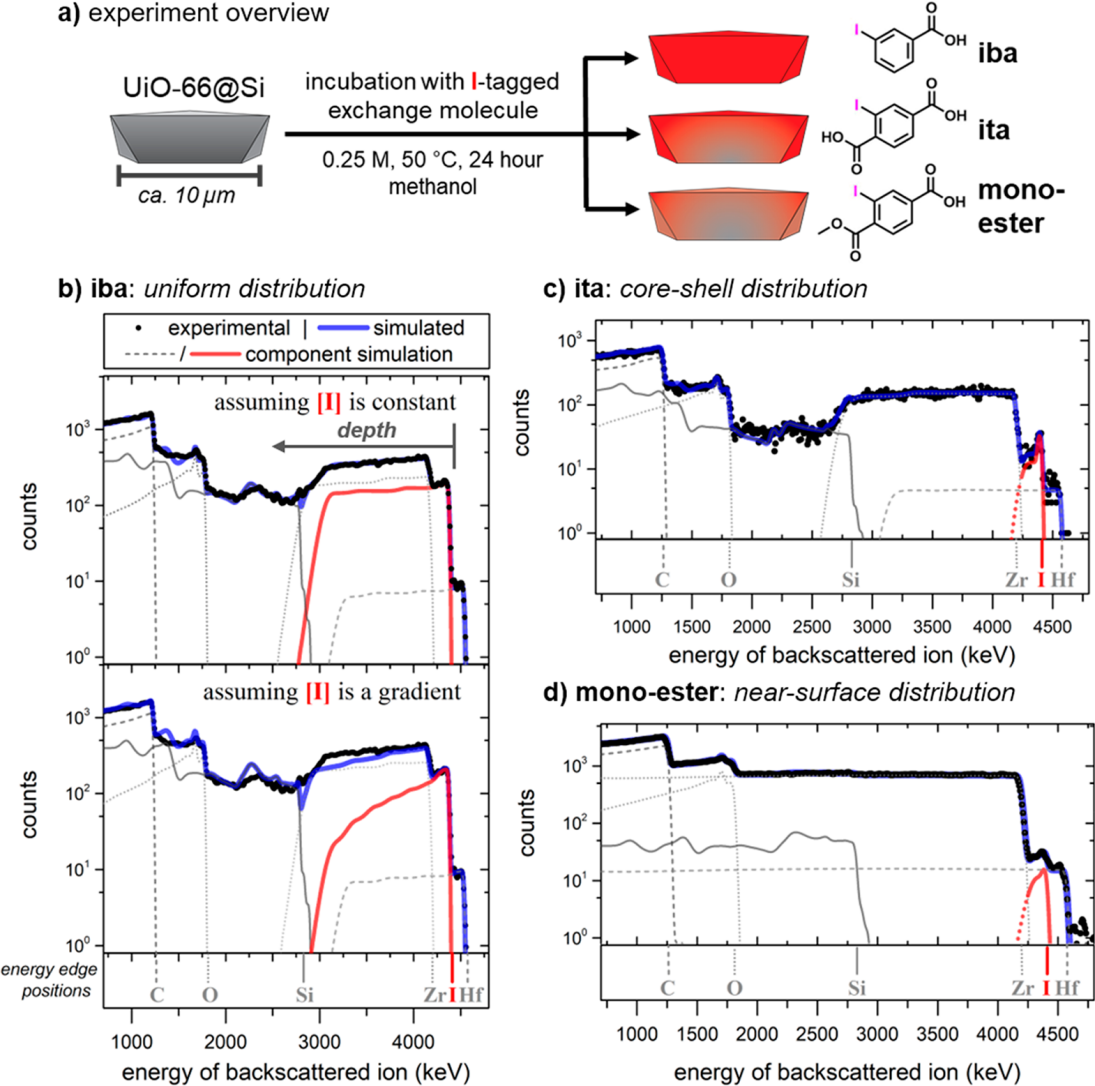
(a) Overview of the postsynthetic exchange process and exchange molecules studied, along with the three outcomes of incorporation. (b–d) Experimental energy spectra and simulation fits of 5 MeV primary He ions backscattered from UiO-66@Si subjected to exchange conditions with (b) 3-iodobenzoic acid (UiO-66-iba) with the top simulated fit assuming a uniform distribution of the incorporated iodine (and, by proxy, the linker) and the bottom simulated fit assuming a decreasing linear gradient of iodine deeper into the crystal, with the poorer fit unambiguously demonstrating the absence of strong concentration dependence on the probed depth; (c) iodoterephthalic acid (UiO-66-ita), displaying a core–shell microstructure with a high near-surface iodine concentration; and (d) 3-iodo-4-(methoxycarbonyl)benzoic acid (UiO-66-monoester) showing a low concentration of iodine near the surface.

Initially, we focused on establishing the reproducibility of the ion microbeam method by repeating measurements of UiO-66-ita and UiO-66-iba on two batches and at least for two crystals in each batch. Initial experiments discovered that, for observable iodine to be incorporated, the UiO-66@Si samples had to be pre-evacuated and soaked in exchange solutions (see Table S1 for optimization results). Representative spectra are shown in Figure 5 together with simulations using SIMNRA.^50^ Signal profiles of zirconium, carbon, oxygen, and silicon (the latter originating from the substrate) were practically identical in the two spectra and indicate homogeneous concentrations, whereas the iodine signals display notable differences. Note that a small amount of hafnium is always observed in the Zr containing MOFs, as hafnium impurities are omnipresent in commercial zirconium due to difficulties of separation.^52^

After PSE of UiO-66@Si with iba, iodine was found uniformly distributed with a constant ratio of I to Zr of 0.44:1 (Figure 5b). To assess which linkers were replaced, bulk UiO-66 and UiO-66-iba were prepared using the same procedure as that for the @Si samples and digested for ^1^H NMR analysis (see the Supporting Information for details). The pristine UiO-66 was found to have a ratio of 5 terephthalates (ta) to 2 formate (fa) molecules (Figure S10). If there are no missing cluster defects, this corresponds to an idealized chemical formula for pristine UiO-66 with this preparation of Zr_6_O_4_(OH)_4_(ta)_5_(fa)_2_. After PSE with iba, ^1^H NMR analysis revealed a ratio of 0.38 iba to terephthalate (Figure S11), nearly identical to the 0.4 ratio expected if 100% of the formates were exchanged with iba. As reported, PSE in UiO-66 proceeds first by exchange of formate before replacement of the original (here, those without iodine) terephthalate linkers.^32^ The ratio of iodine to Zr found by RBS (0.44) is higher than the idealized ratio estimated by ^1^H NMR (0.33), with the difference likely explained by defects,^32^ and discrepancies between bulk and @SI prepared samples.

To illustrate the necessity of a homogeneous iodine concentration in order to achieve a good fit to the experimental data, we also simulated a linear gradient in iodine concentration (lower panel of Figure 5b), which resulted in a visibly poorer fit. As a constant concentration ratio between Zr and I leads to an excellent fit and as the cross sections for iodine and zirconium are not identical, any meaningful gradient with decreasing iodine concentration would lead to a worse fit. The ratio of Zr:I as a function of depth for the two fits is shown in Figure S6.

Uniformly distributed iba can be rationalized by a sufficiently fast diffusion rate. This diffusion rate depends on multiple factors, including size, the number of carboxylates in the molecule, and their p*K*_a_. Previous SALE experiments showed that modulators can quantitatively be substituted by ligands with a lower p*K*_a_, while incoming ligands with a higher p*K*_a_ than the SBU-bound modulator are not incorporated.^53^ Zhang et al. rationalized this in terms of carboxylic acids with lower p*K*_a_’s being more competitive for node ligation compared to those with a higher p*K*_a_.^54^ Competing with this effect, however, is that, once formed, the ligand–SBU bond strength increases with decreasing p*K*_a_ of the ligand.^55,56^ Applying this logic to the present case, iba (p*K*_a_ = 3.87)^57^ likely exists less in the deprotonated state compared to ita (1st p*K*_a_ = 2.45 ± 0.01, calcd),^58^ which should render iba a more quickly diffusing species inside UiO-66, in addition to being physically smaller than ita. However, it should be noted that these are aqueous p*K*_a_’s; given that relative p*K*_a_’s can switch order in non-aqueous solvents and that protonation in non-aqueous solvents is complex,^59^ it is likely that the pronation dynamics are more complex than discussed here. Photometric titrations assessing the average p*K*_a_ of the crystals and the bulk solution, both before and after PSE, could provide deeper insights, though developing those methods was beyond the scope of this work.

Indeed, in contrast to the PSE experiments with iba, subjecting ita to the sample led to a high iodine concentration near the crystal surface with a rapidly decaying concentration deeper into the bulk crystal (Figure 5c). The rest of the spectrum resembles that of a crystal prior to PSE. Specifically, only in a layer with a thickness of approximately 0.2 μm, the I:Zr ratio is found to be as high as 0.14:1. After the first micrometer from the surface, the concentration drops to at most 0.05:1 with maximum bulk concentrations being below 0.03:1. While an exact quantification of a potential incorporated iodine deep inside the crystal at concentrations below this limit is hampered by the overlap with the dominant Zr signal (mind the logarithmic scale), from the observed decrease of the near-surface signal, even lower concentrations can be anticipated.

In a previous study on PSE of ita into thin films of submicrometer sized UiO-66 on Si using bulk analysis RBS (rather than the microbeam used here),^28,45^ we demonstrated a uniform ita distribution throughout the crystals, even for exchange processes as short as a few seconds. Prolonged exposure to the PSE solution resulted in a higher incorporation yield but did not change the ita distribution in the crystal. Compared to these earlier findings, the core–shell distribution found for the ∼15 μm sized UiO-66 single crystals described herein is intriguing but not unexpected. It can be explained by a model that relates the PSE distribution to the relative kinetics of the diffusion of incoming molecules in the crystal bulk and the actual ligand exchange.^27^

If crystals are similar in size or smaller than the distance traveled by the diffusion front over the course of the PSE experiment, diffusion limitations can be neglected and the exchange process will occur uniformly throughout the crystal (for a more thorough discussion of intra-MOF diffusion, please see refs (10 and 60−63)). In addition, in a bulk experiment with submicrometer sized crystals, incoming linkers can enter the crystal from all sides, further decreasing transport limitations. We believe this accounts for the uniform distribution of ita observed in submicrometer sized UiO-66.^28,45^ The core–shell structure observed herein indicates that the diffusion front of ita after 24 h of incubation does not reach deeper than ∼0.2 μm into the UiO-66 crystal and diffusion can be considered a quasi-1D process. Notably, the **ita** shell as detected by RBS does not have a sharp boundary, but the iodine content decays exponentially beyond 0.2 μm. The term “core–shell” is thus more colloquial rather than strictly accurate, as recently also pointed out by Matzger and co-workers during studies on PSE in MOF-5.^27^

As the size of the crystal greatly exceeds the thickness of the shell, the order of magnitude of the apparent diffusion coefficient for ita diffusion can be estimated to *D* ≈ 10^–19^ m^2^/s assuming diffusion as a pseudo-1D process (see the Supporting Information). For comparison, a series of amines diffusing into solvent-filled MOF-1 pores displayed diffusion coefficients ranging from ∼10^–14^ to ∼10^–13^ m^2^/s.^64^ We note that, for a complete kinetic description of the PSE process, diffusion of the detached native MOF species (linker and modulator) would also need to be considered, as they continue to compete for node ligation while being in the bulk of the crystal.

The presence of unprotected carboxylic acid groups in the exchanging linkers is a crucial factor that slows down diffusion rates, as also reported by Matzger and co-workers. While the methyl esters of *d*^4^-teraphthalic acid and *d*^5^-benzoic acid diffused much faster through large (>100 μm) MOF-5 crystals, the free acid forms of both ligands formed core–shell structures.^27^ In the UiO-66 crystals studied here, one free carboxylic acid in iba does not sufficiently slow down diffusion, and it is only with two of these carboxylates that a core–shell structure is observed.

To test this hypothesis as well as the impact of molecular size, an ion microbeam was used to analyze the distribution of the **monoester** of ita after PSE into UiO-66 to yield UiO-66-**monoester**. The **monoester** is sterically larger than **ita** but has one fewer free carboxylic acid group. Microbeam analysis (Figure 5d) revealed that the **monoester** was observed near the crystal surface (to a depth of ca. 0.2 μm) in a concentration of about 1 **monoester** to 70 Zr. Similar as for the ita samples, a gradient indicating further decreasing iodine concentrations toward the bulk can be observed. Again, a potential, though very low concentration (ca. 1 I to 200 Zr) extending all over the bulk of the crystal cannot be fully excluded, but is not anticipated from the data at hand. This finding is in contrast to the work by Matzger, who found deeper incorporation of the methyl ester of benzoic acid.^27^ We hypothesize that in the case of the **monoester** the large size of the iodine substituent counteracts the effect of having one fewer carboxylic group.

### Spatial Distribution of Postsynthetic Metalation

As a final example to demonstrate the utility of an ion microbeam to obtain depth profiles, we sought to measure the distribution of metal sites within single MOF crystals after postsynthetic metalation (PSM). PSM has been widely employed to add functionality to MOFs, especially to create site-isolated single-metal catalytic sites.^23,24^ This has particular relevance because, while homogeneous molecular catalysts are powerful, they are rarely of sufficient *stability* and technological readiness. As a common decomposition pathway for molecular catalysis is via bimolecular reactions, fixing individual catalyst units in a porous support can improve their long-term stability. UiO-67-bpy, a MOF in which the biphenyl linkers of the parent UiO-67 are replaced with some fraction—up to 100%^23^—with bipyridine linkers is a common platform for PSM.^24^ However, to our knowledge, only one report exists which investigated the depth distribution of metalated sites within UiO-67-bpy.^26^

**Figure 6:**
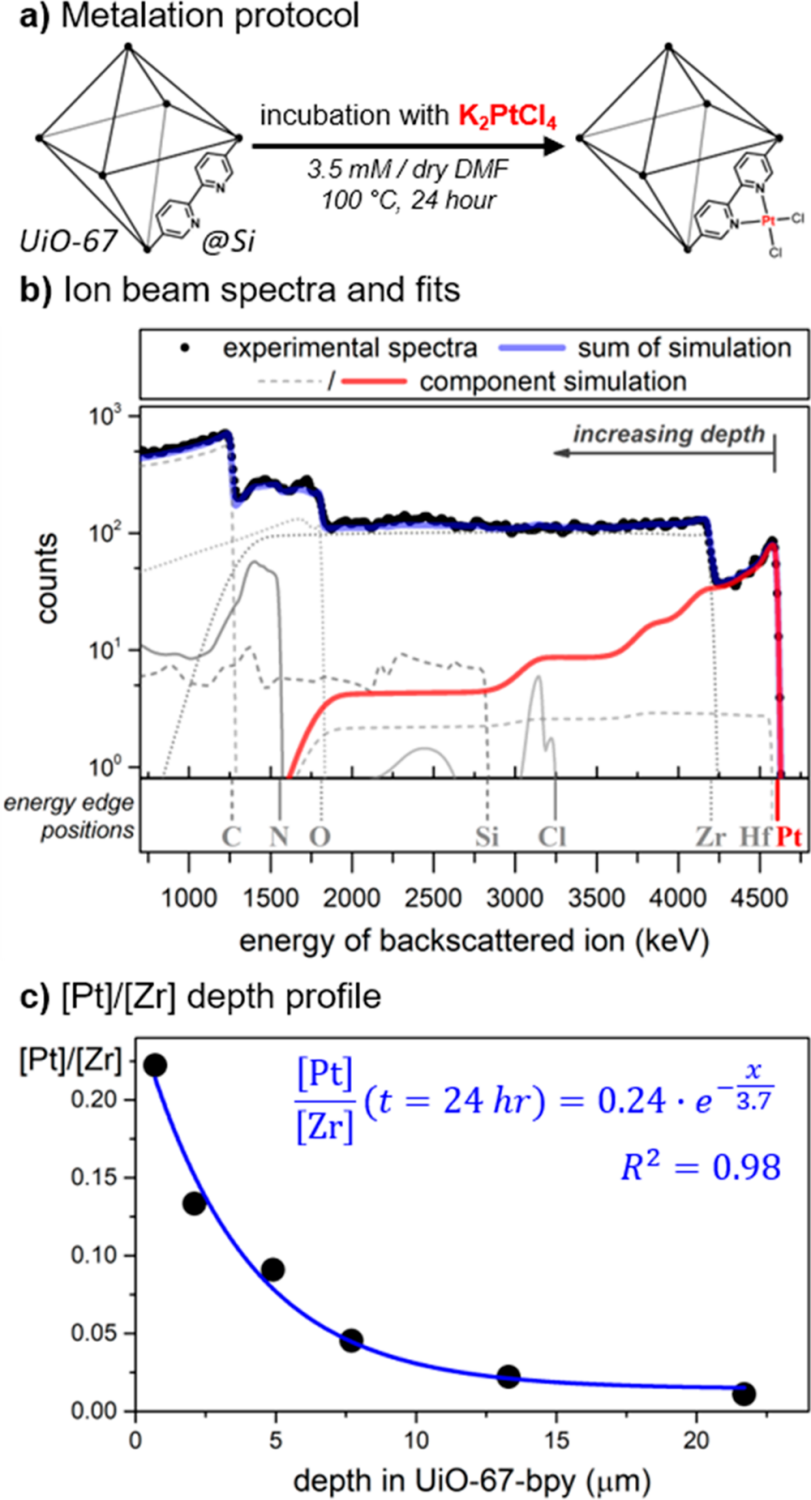
(a) Metalation protocol of UiO-67-bpy@Si crystals, (b) ion beam spectra of UiO-67-bpy after metalation with K_2_PtCl_4_, and (c) ratio of [Pt] to [Zr] as a function of depth in UiO-67-bpy after metalation, where a ratio of 1 would indicate 100% metalation of all bipyridine sites.

Isolated single crystals of UiO-67-bpy on silicon slides were synthesized using a modification of a method reported by Long and co-workers, evacuated, incubated with K_2_PtCl_4_, and washed extensively (see Figure 6a and the Supporting Information for full details).^65^ PXRD of the resulting slides confirmed that the material had the typical UiO-67 reflections (Figure S7). By SEM, the crystals were approximately 20 μm in size and generally well isolated from one another (Figure S8). For microbeam analysis, a crystal oriented with the ⟨111⟩ plane parallel to the surface was chosen.

As shown in Figure 6b, the backscattering spectra show the presence of all expected elements, with a strong Pt signal that decays as a function of crystal depth. Notably, no potassium was observed, indicative that the concentration of trapped K_2_PtCl_4_ was less than 1%. The ratio of Pt to Cl was modeled as 1:2, though we should note that the weakness of the chlorine signal means that different ratios could be used without noticeably impacting the fit. Meanwhile, the ratio of Zr to N was 1:1.2, very close to the expected stoichiometry of 1:2.

The ratio of Pt to Zr near the crystal surface was ca. 0.25, suggesting that only a quarter of the bipyridine sites at the surface had been metalated. The concentration of Pt then decays deeper into the crystal at an exponential rate, as shown in Figure 6c, though some Pt is present at least up to 12 μm deep. Intriguingly, for single crystals of UiO-67-bpy ranging from ca. 40 to 100 μm in size, complete metalation using Cu^II^, Cu^I^, Co^II^, Fe^II^, and Cr^II^ has been reported as verified by single crystal structure analysis^65^—a contrast to the clear gradient of metalation observed here for 20 μm sized crystals metalated with Pt. We believe this difference points primarily to the difference in incubation time: while the crystals reported to be exhaustively metalated were incubated for 7 days, the sample measured here was incubated for 1 day. In addition, when crystals are mounted on a Si slide, a metal precursor can diffuse into fewer of the crystal faces.

Furthermore, accumulating evidence strongly suggests that UiO-67 and UiO-67-bpy especially are quite moisture sensitive—a clear contrast to the often-reported claim that the whole UiO MOF series is water stable.^66^ As UiO-67-bpy samples were prepared outside of a glovebox, it is possible that degradation of the Zr SBUs results in surface barriers which inhibit uptake of the Pt^II^ precursor. Indeed, PXRD of the samples post metalation showed decreased crystallinity (Figure S9). We are currently investigating these possibilities and plan to use the ion microbeam analysis to assess the impact of sample preparation on metalation depth distribution.

The data shown in Figure 6c essentially provides a snapshot in time of metalation; concentration profiles like this one can be used to estimate diffusivity constants starting from Fick’s laws.^35,67^ Such analysis has been performed for porous materials, for example, on methanol diffusion through porous materials, and exponential-like profiles were observed.^68^ A model to adequately describe the concentration profile of Figure 6c would require knowing the surface permittivity of UiO-67 (which would include the kinetics associated with surface barriers^60^) and the equilibrium binding kinetics of the Pt precursor to the bipyridine sites. In addition, since every successful metalation event yields a more sterically hindered pore environment, the diffusivity constant of the Pt precursor likely varies as a function of both time and space. To our knowledge, no such model has yet been constructed for MOFs; consequently, the data obtainable by ion microbeam for postsynthetic metalation suggests a promising area of research.

## Conclusions

In summary, we have demonstrated that an ion microbeam can be used to nondestructively obtain ca. 100 and 150 nm resolution elemental depth profiles of intact UiO-66 and UiO-67-bpy MOF single crystals, respectively. Raster scanning of the crystals was found to be necessary to avoid thermal damage from the microbeam. The method was first used to probe the distribution of a mixed-metal Zr/Hf UiO-66 MOF, showing that for this synthetic protocol Zr and Hf were evenly distributed. Next, the depth profiles of UiO-type crystals after either postsynthetic linker exchange or metalation were determined. A strong dependence of the depth profile on the number of carboxylic acid groups was observed, where mono carboxylic acid molecules resulted in a uniform intra-MOF distribution, whereas the dicarboxylic acid exchange molecule resulted in a clear core–shell distribution. Metalation of UiO-67-bpy with PtCl_2_ resulted again in a core–shell material but with a greater penetration depth of Pt (up to tens of microns).

The sensitivity and local character of microbeam backscattering spectrometry applied to highly oriented, surface grown MOF single crystals represents a powerful platform to investigate a variety of otherwise inaccessible transport phenomena as pseudo-1D systems. While the present work focuses on the depth profiling of postsynthetically modified sites, the method can easily be extended to studies on the spatial distribution of metal exchanges at SBUs, as shown with the initial Hf data, a strategy that holds great promise for catalysis.^69−71^ With the field of MOF–catalyst composites quickly growing, knowledge of the spatial distribution of catalytic species within the MOFs is crucial for catalyst benchmarking^10^ and needs to be addressed with robust and accurate methods.

It should be noted that herein we focused on analyzing elements with higher mass than Zr, as it provided quicker insight for method development. To reach its full potential, microbeam analysis must be applicable for the depth analysis of lighter elements. Ongoing work in our laboratories has begun in this direction. As an example, combination with nuclear reaction analysis probing specific isotopes of light elements used for isotopic labeling such as D, ^15^N, ^18^O, or similar can be attractive, in particular when employing a probing beam of protons, deuterium, or ^3^He. In future measurements, bulk crystal analysis by particle induced X-ray emission (PIXE) providing integral yields but limited depth information in combination with the present approach providing depth profiles with highest sensitivity to near-surface regions can further expand the capabilities of the method.

We believe that the microbeam approach is a significant step forward in depth-resolved elemental mapping of intact MOF single crystals and will thus aid in untapping the full potential that is offered by molecular modifications of heterogeneous 3D materials.

## Supporting Information Available

Experimental details including details on the nuclear microbeam experiment, syntheses, ^1^H NMR spectra of digested MOFs, SEM images, PXRD patterns, and estimation of the apparent diffusion coefficient (PDF)

## References

[ref1] Wang H.-F.; Chen L.; Pang H.; Kaskel S.; Xu Q. MOF-Derived Electrocatalysts for Oxygen Reduction, Oxygen Evolution and Hydrogen Evolution Reactions. Chem. Soc. Rev. 2020, 49 (5), 1414–1448. 10.1039/C9CS00906J. 32039429

[ref2] Liao P.-Q.; Shen J.-Q.; Zhang J.-P. Metal–Organic Frameworks for Electrocatalysis. Coord. Chem. Rev. 2018, 373, 22–48. 10.1016/j.ccr.2017.09.001.

[ref3] Kornienko N.; Zhao Y.; Kley C. S.; Zhu C.; Kim D.; Lin S.; Chang C. J.; Yaghi O. M.; Yang P. Metal–Organic Frameworks for Electrocatalytic Reduction of Carbon Dioxide. J. Am. Chem. Soc. 2015, 137 (44), 14129–14135. 10.1021/jacs.5b08212. 26509213

[ref4] Lin S.; Diercks C. S.; Zhang Y.-B.; Kornienko N.; Nichols E. M.; Zhao Y.; Paris A. R.; Kim D.; Yang P.; Yaghi O. M.; Chang C. J. Covalent Organic Frameworks Comprising Cobalt Porphyrins for Catalytic CO 2 Reduction in Water. Science 2015, 349 (6253), 1208–1213. 10.1126/science.aac8343. 26292706

[ref5] Lin S.; Ravari A. K.; Zhu J.; Usov P. M.; Cai M.; Ahrenholtz S. R.; Pushkar Y.; Morris A. J. Insight into Metal-Organic Framework Reactivity: Chemical Water Oxidation Catalyzed by a [Ru(Tpy)(Dcbpy)(OH 2)] 2+ -Modified UiO-67. ChemSusChem 2018, 11 (2), 464–471. 10.1002/cssc.201701644. 29197150

[ref6] Hod I.; Sampson M. D.; Deria P.; Kubiak C. P.; Farha O. K.; Hupp J. T. Fe-Porphyrin-Based Metal–Organic Framework Films as High-Surface Concentration, Heterogeneous Catalysts for Electrochemical Reduction of CO_2_. ACS Catal. 2015, 5 (11), 6302–6309. 10.1021/acscatal.5b01767.

[ref7] Kaeffer N.; Morozan A.; Fize J.; Martinez E.; Guetaz L.; Artero V. The Dark Side of Molecular Catalysis: Diimine-Dioxime Cobalt Complexes Are Not the Actual Hydrogen Evolution Electrocatalyst in Acidic Aqueous Solutions. ACS Catal. 2016, 6 (6), 3727–3737. 10.1021/acscatal.6b00378.

[ref8] Johnson B. A.; Bhunia A.; Fei H.; Cohen S. M.; Ott S. Development of a UiO-Type Thin Film Electrocatalysis Platform with Redox-Active Linkers. J. Am. Chem. Soc. 2018, 140 (8), 2985–2994. 10.1021/jacs.7b13077. 29421875 PMC6067658

[ref9] Roy S.; Huang Z.; Bhunia A.; Castner A.; Gupta A. K.; Zou X.; Ott S. Electrocatalytic Hydrogen Evolution from a Cobaloxime-Based Metal–Organic Framework Thin Film. J. Am. Chem. Soc. 2019, 141 (40), 15942–15950. 10.1021/jacs.9b07084. 31508946 PMC6803166

[ref10] Johnson B. A.; Beiler A. M.; McCarthy B. D.; Ott S. Transport Phenomena: Challenges and Opportunities for Molecular Catalysis in Metal–Organic Frameworks. J. Am. Chem. Soc. 2020, 142 (28), 11941–11956. 10.1021/jacs.0c02899. 32516534 PMC7366383

[ref11] Yang D.; Gates B. C. Catalysis by Metal Organic Frameworks: Perspective and Suggestions for Future Research. ACS Catal. 2019, 9 (3), 1779–1798. 10.1021/acscatal.8b04515.

[ref12] Budnikova Y. H. Recent Advances in Metal-Organic Frameworks for Electrocatalytic Hydrogen Evolution and Overall Water Splitting Reactions. Dalt. Trans. 2020, 49 (36), 12483–12502. 10.1039/D0DT01741H. 32756705

[ref13] Bavykina A.; Kolobov N.; Khan I. S.; Bau J. A.; Ramirez A.; Gascon J. Metal-Organic Frameworks in Heterogeneous Catalysis: Recent Progress, New Trends, and Future Perspectives. Chem. Rev. 2020, 120 (16), 8468–8535. 10.1021/acs.chemrev.9b00685. 32223183

[ref14] Downes C. A.; Marinescu S. C. Electrocatalytic Metal-Organic Frameworks for Energy Applications. ChemSusChem 2017, 10 (22), 4374–4392. 10.1002/cssc.201701420. 28968485

[ref15] McCarthy B. D.; Beiler A. M.; Johnson B. A.; Liseev T.; Castner A. T.; Ott S. Analysis of Electrocatalytic Metal-Organic Frameworks. Coord. Chem. Rev. 2020, 406, 213137 10.1016/j.ccr.2019.213137. 32499663 PMC7272229

[ref16] Eddaoudi M.; Kim J.; Rosi N.; Vodak D.; Wachter J.; O’Keefe M.; Yaghi O. M. Systematic Design of Pore Size and Functionality in Isoreticular MOFs and Their Application in Methane Storage. Science 2002, 295 (5554), 469–472. 10.1126/science.1067208. 11799235

[ref17] Kirchon A.; Feng L.; Drake H. F.; Joseph E. A.; Zhou H.-C. From Fundamentals to Applications: A Toolbox for Robust and Multifunctional MOF Materials. Chem. Soc. Rev. 2018, 47 (23), 8611–8638. 10.1039/C8CS00688A. 30234863

[ref18] Evans J. D.; Sumby C. J.; Doonan C. J. Post-Synthetic Metalation of Metal-Organic Frameworks. Chem. Soc. Rev. 2014, 43 (16), 5933–5951. 10.1039/C4CS00076E. 24736674

[ref19] Kim M.; Cahill J. F.; Su Y.; Prather K. A.; Cohen S. M. Postsynthetic Ligand Exchange as a Route to Functionalization of ‘Inert’ Metal–Organic Frameworks. Chem. Sci. 2012, 3 (1), 126–130. 10.1039/C1SC00394A.

[ref20] Karagiaridi O.; Bury W.; Mondloch J. E.; Hupp J. T.; Farha O. K. Solvent-Assisted Linker Exchange: An Alternative to the De Novo Synthesis of Unattainable Metal-Organic Frameworks. Angew. Chem., Int. Ed. 2014, 53 (18), 4530–4540. 10.1002/anie.201306923. 24652755

[ref21] Cohen S. M. The Postsynthetic Renaissance in Porous Solids. J. Am. Chem. Soc. 2017, 139 (8), 2855–2863. 10.1021/jacs.6b11259. 28118009

[ref22] Marshall R. J.; Forgan R. S. Postsynthetic Modification of Zirconium Metal-Organic Frameworks. Eur. J. Inorg. Chem. 2016, 2016 (27), 4310–4331. 10.1002/ejic.201600394.

[ref23] Fei H.; Cohen S. M. A Robust, Catalytic Metal–Organic Framework with Open 2,2′-Bipyridine Sites. Chem. Commun. 2014, 50 (37), 4810–4812. 10.1039/C4CC01607F. 24687158

[ref24] Tu T. N.; Nguyen M. V.; Nguyen H. L.; Yuliarto B.; Cordova K. E.; Demir S. Designing Bipyridine-Functionalized Zirconium Metal–Organic Frameworks as a Platform for Clean Energy and Other Emerging Applications. Coord. Chem. Rev. 2018, 364, 33–50. 10.1016/j.ccr.2018.03.014.

[ref25] Ji Z.; Li T.; Yaghi O. M. Sequencing of Metals in Multivariate Metal-Organic Frameworks. Science 2020, 369 (6504), 674–680. 10.1126/science.aaz4304. 32764067

[ref26] Luo T.-Y.; Liu C.; Gan X. Y.; Muldoon P. F.; Diemler N. A.; Millstone J. E.; Rosi N. L. Multivariate Stratified Metal-Organic Frameworks: Diversification Using Domain Building Blocks. J. Am. Chem. Soc. 2019, 141 (5), 2161–2168. 10.1021/jacs.8b13502. 30636428

[ref27] Boissonnault J. A.; Wong-Foy A. G.; Matzger A. J. Core–Shell Structures Arise Naturally During Ligand Exchange in Metal–Organic Frameworks. J. Am. Chem. Soc. 2017, 139 (42), 14841–14844. 10.1021/jacs.7b08349. 29020774

[ref28] Fluch U.; Paneta V.; Primetzhofer D.; Ott S. Uniform Distribution of Post-Synthetic Linker Exchange in Metal–Organic Frameworks Revealed by Rutherford Backscattering Spectrometry. Chem. Commun. 2017, 53 (48), 6516–6519. 10.1039/C7CC02631E. PMC5846729 28573305

[ref29] Kim S.; Lee J.; Jeoung S.; Moon H. R.; Kim M. Surface-Deactivated Core–Shell Metal–Organic Framework by Simple Ligand Exchange for Enhanced Size Discrimination in Aerobic Oxidation of Alcohols.. Chem. - Eur. J. 2020, 26 (34), 7568–7572. 10.1002/chem.202000933. 32096306

[ref30] Pereira C. F.; Howarth A. J.; Vermeulen N. A.; Almeida Paz F. A.; Tomé J. P. C.; Hupp J. T.; Farha O. K. Towards Hydroxamic Acid Linked Zirconium Metal-Organic Frameworks. Mater. Chem. Front. 2017, 1 (6), 1194–1199. 10.1039/C6QM00364H.

[ref31] Marreiros J.; Caratelli C.; Hajek J.; Krajnc A.; Fleury G.; Bueken B.; De Vos D. E.; Mali G.; Roeffaers M. B. J.; Van Speybroeck V.; Ameloot R. Active Role of Methanol in Post-Synthetic Linker Exchange in the Metal–Organic Framework UiO-66. Chem. Mater. 2019, 31 (4), 1359–1369. 10.1021/acs.chemmater.8b04734.

[ref32] Taddei M.; Wakeham R. J.; Koutsianos A.; Andreoli E.; Barron A. R. Post-Synthetic Ligand Exchange in Zirconium-Based Metal-Organic Frameworks: Beware of The Defects! *Angew*. Angew. Chem., Int. Ed. 2018, 57 (36), 11706–11710. 10.1002/anie.201806910. 29989290

[ref33] Zhu J.; Maza W. A.; Morris A. J. Light-Harvesting and Energy Transfer in Ruthenium(II)-Polypyridyl Doped Zirconium(IV) Metal-Organic Frameworks: A Look toward Solar Cell Applications. J. Photochem. Photobiol., A 2017, 344 (C), 64–77. 10.1016/j.jphotochem.2017.04.025.

[ref34] Dodson R. A.; Kalenak A. P.; Matzger A. J. Solvent Choice in Metal–Organic Framework Linker Exchange Permits Microstructural Control. J. Am. Chem. Soc. 2020, 142 (49), 20806–20813. 10.1021/jacs.0c10224. 33237750

[ref35] Heinke L.; Kärger J. Assessing One-Dimensional Diffusion in Nanoporous Materials from Transient Concentration Profiles. New J. Phys. 2008, 10 (2), 023035 10.1088/1367-2630/10/2/023035.

[ref36] Liu C.; Zeng C.; Luo T.-Y.; Merg A. D.; Jin R.; Rosi N. L. Establishing Porosity Gradients within Metal-Organic Frameworks Using Partial Postsynthetic Ligand Exchange. J. Am. Chem. Soc. 2016, 138 (37), 12045–12048. 10.1021/jacs.6b07445. 27593173

[ref37] Jayachandrababu K. C.; Sholl D. S.; Nair S. Structural and Mechanistic Differences in Mixed-Linker Zeolitic Imidazolate Framework Synthesis by Solvent Assisted Linker Exchange and de Novo Routes. J. Am. Chem. Soc. 2017, 139 (16), 5906–5915. 10.1021/jacs.7b01660. 28388071

[ref38] Geiger H.; Marsden E. The Laws of Deflexion of α Particles through Large Angles.. London, Edinburgh, Dublin Philos. Mag. J. Sci. 1913, 25 (148), 604–623. 10.1080/14786440408634197.

[ref39] Rutherford E. The Scattering of α and β Particles by Matter and the Structure of the Atom. London, Edinburgh, Dublin Philos. Mag. J. Sci. 1911, 21 (125), 669–688. 10.1080/14786440508637080.

[ref40] Bergmann U.; Apelt S.; Khojasteh N. B.; Heller R. Solid–Liquid Interface Analysis with In-situ Rutherford Backscattering and Electrochemical Impedance Spectroscopy. Surf. Interface Anal. 2020, 52 (12), 1111–1116. 10.1002/sia.6835.

[ref41] Vadrucci M.; Mazzinghi A.; Sorrentino B.; Falzone S.; Gioia C.; Gioia P.; Loreti E. M.; Chiari M. Characterisation of Ancient Roman Wall-painting Fragments Using Non-destructive IBA and MA-XRF Techniques. X-Ray Spectrom. 2020, 49 (6), 668–678. 10.1002/xrs.3178.

[ref42] Vilarigues M.; da Silva R. C. Ion Beam and Infrared Analysis of Medieval Stained Glass. Appl. Phys. A: Mater. Sci. Process. 2004, 79 (2), 373–378. 10.1007/s00339-004-2538-9.

[ref43] Santos H. C.; Added N.; Silva T. F.; Rodrigues C. L. External-RBS, PIXE and NRA Analysis for Ancient Swords. Nucl. Instrum. Methods Phys. Res., Sect. B 2015, 345, 42–47. 10.1016/j.nimb.2014.12.076.

[ref44] Wagner A.; Pullen S.; Ott S.; Primetzhofer D. The Potential of Ion Beams for Characterization of Metal-Organic Frameworks.. Nucl. Instrum. Methods Phys. Res., Sect. B 2016, 371, 327–331. 10.1016/j.nimb.2015.10.059.

[ref45] Paneta V.; Fluch U.; Petersson P.; Ott S.; Primetzhofer D. Characterization of Compositional Modifications in Metal-Organic Frameworks Using Carbon and Alpha Particle Microbeams.. Nucl. Instrum. Methods Phys. Res., Sect. B 2017, 404, 198–201. 10.1016/j.nimb.2017.01.058.

[ref46] Handbook of Modern Ion Beam Materials Analysis; Nastasi M. A., Tesmer J. R., Eds.; Materials Research Society: 2009.

[ref47] Abednatanzi S.; Derakhshandeh P. G.; Depauw H.; Coudert F.-X.; Vrielinck H.; Van Der Voort P.; Leus K. Mixed-Metal Metal–Organic Frameworks. Chem. Soc. Rev. 2019, 48 (9), 2535–2565. 10.1039/C8CS00337H. 30989162

[ref48] Cotton S. A.; Hart F. A. Zirconium and Hafnium. In The Heavy Transition Elements; Macmillan Education UK: London, 1975; pp 3–14. 10.1007/978-1-349-15591-0_1.

[ref49] Depauw H.; Nevjestić I.; De Winne J.; Wang G.; Haustraete K.; Leus K.; Verberckmoes A.; Detavernier C.; Callens F.; De Canck E.; Vrielinck H.; Van Der Voort P. Microwave Induced “Egg Yolk” Structure in Cr/V-MIL-53. Chem. Commun. 2017, 53 (60), 8478–8481. 10.1039/C7CC04651K. 28703241

[ref50] Mayer M. Improved Physics in SIMNRA 7. Nucl. Instrum. Methods Phys. Res., Sect. B 2014, 332, 176–180. 10.1016/j.nimb.2014.02.056.

[ref51] Lomachenko K. A.; Jacobsen J.; Bugaev A. L.; Atzori C.; Bonino F.; Bordiga S.; Stock N.; Lamberti C. Exact Stoichiometry of Ce x Zr 6– x Cornerstones in Mixed-Metal UiO-66 Metal–Organic Frameworks Revealed by Extended X-Ray Absorption Fine Structure Spectroscopy. J. Am. Chem. Soc. 2018, 140 (50), 17379–17383. 10.1021/jacs.8b10343. 30497258

[ref52] Cox R. P.; Peterson H. C.; Beyer G. H. Separating Hafnium from Zirconium. Solvent Extraction with Tributyl Phosphate. Ind. Eng. Chem. 1958, 50 (2), 141–143. 10.1021/ie50578a022.

[ref53] Deria P.; Bury W.; Hupp J. T.; Farha O. K. Versatile Functionalization of the Nu-1000 Platform by Solvent-Assisted Ligand Incorporation. Chem. Commun. 2014, 50 (16), 1965–1968. 10.1039/c3cc48562e. 24406797

[ref54] Zhang W.; Bu A.; Ji Q.; Min L.; Zhao S.; Wang Y.; Chen J. P Ka-Directed Incorporation of Phosphonates into MOF-808 via Ligand Exchange: Stability and Adsorption Properties for Uranium. ACS Appl. Mater. Interfaces 2019, 11 (37), 33931–33940. 10.1021/acsami.9b10920. 31409065

[ref55] Karagiaridi O.; Bury W.; Tylianakis E.; Sarjeant A. A.; Hupp J. T.; Farha O. K. Opening Metal–Organic Frameworks Vol. 2: Inserting Longer Pillars into Pillared-Paddlewheel Structures through Solvent-Assisted Linker Exchange. Chem. Mater. 2013, 25 (17), 3499–3503. 10.1021/cm401724v.

[ref56] Deria P.; Mondloch J. E.; Karagiaridi O.; Bury W.; Hupp J. T.; Farha O. K. Beyond Post-Synthesis Modification: Evolution of Metal–Organic Frameworks via Building Block Replacement. Chem. Soc. Rev. 2014, 43 (16), 5896–5912. 10.1039/C4CS00067F. 24723093

[ref57] CRC Handbook of Chemistry and Physics, 100th ed.; Rumble J. R., Ed.; CRC Press, Taylor & Francis Group: 2019.

[ref58] Advanced Chemistry Development Software. Calculated by Advanced Chemistry Development Software by ACD/Labs 2020.

[ref59] Reed C. A. Myths about the Proton. The Nature of H(+) in Condensed Media. Acc. Chem. Res. 2013, 46 (11), 2567–2575. 10.1021/ar400064q. 23875729 PMC3833890

[ref60] Heinke L.; Gu Z.; Wöll C. The Surface Barrier Phenomenon at the Loading of Metal-Organic Frameworks. Nat. Commun. 2014, 5 (May), 1–6. 10.1038/ncomms5562. 25078573

[ref61] Celis-Salazar P. J.; Cai M.; Cucinell C. A.; Ahrenholtz S. R.; Epley C. C.; Usov P. M.; Morris A. J. Independent Quantification of Electron and Ion Diffusion in Metallocene-Doped Metal–Organic Frameworks Thin Films. J. Am. Chem. Soc. 2019, 141 (30), 11947–11953. 10.1021/jacs.9b03609. 31271285

[ref62] Cai M.; Loague Q.; Morris A. J. Design Rules for Efficient Charge Transfer in Metal–Organic Framework Films: The Pore Size Effect. J. Phys. Chem. Lett. 2020, 11, 702–709. 10.1021/acs.jpclett.9b03285. 31917577

[ref63] Johnson B. A. Interrogating Diffusional Mass and Charge Transport in Catalytic Metal-Organic Frameworks; 2020.

[ref64] Wang C.; Lin W. Diffusion-Controlled Luminescence Quenching in Metal-Organic Frameworks. J. Am. Chem. Soc. 2011, 133 (12), 4232–4235. 10.1021/ja111197d. 21384886

[ref65] Gonzalez M. I.; Bloch E. D.; Mason J. A.; Teat S. J.; Long J. R. Single-Crystal-to-Single-Crystal Metalation of a Metal-Organic Framework: A Route toward Structurally Well-Defined Catalysts. Inorg. Chem. 2015, 54 (6), 2995–3005. 10.1021/acs.inorgchem.5b00096. 25719803

[ref66] Decoste J. B.; Peterson G. W.; Jasuja H.; Glover T. G.; Huang Y. G.; Walton K. S. Stability and Degradation Mechanisms of Metal-Organic Frameworks Containing the Zr6O4(OH)4 Secondary Building Unit. J. Mater. Chem. A 2013, 1 (18), 5642–5650. 10.1039/c3ta10662d.

[ref67] Zheng B.; Huang K.-W.; Du H. Theoretical Model Estimation of Guest Diffusion in Metal–Organic Frameworks (MOFs). RSC Adv. 2015, 5 (86), 70433–70438. 10.1039/C5RA11325C.

[ref68] Heinke L.; Tzoulaki D.; Chmelik C.; Hibbe F.; van Baten J. M.; Lim H.; Li J.; Krishna R.; Kärger J. Assessing Guest Diffusivities in Porous Hosts from Transient Concentration Profiles. Phys. Rev. Lett. 2009, 102 (6), 065901 10.1103/PhysRevLett.102.065901. 19257607

[ref69] Comito R. J.; Fritzsching K. J.; Sundell B. J.; Schmidt-Rohr K.; Dincǎ M. Single-Site Heterogeneous Catalysts for Olefin Polymerization Enabled by Cation Exchange in a Metal-Organic Framework. J. Am. Chem. Soc. 2016, 138 (32), 10232–10237. 10.1021/jacs.6b05200. 27443860

[ref70] Brozek C. K.; Dincă M. Cation Exchange at the Secondary Building Units of Metal–Organic Frameworks. Chem. Soc. Rev. 2014, 43 (16), 5456–5467. 10.1039/C4CS00002A. 24831234

[ref71] Ha J.; Lee J. H.; Moon H. R. Alterations to Secondary Building Units of Metal–Organic Frameworks for the Development of New Functions. Inorg. Chem. Front. 2020, 7 (1), 12–27. 10.1039/C9QI01119F.

