# Supplementary material for: Elemental Depth Profiling of Intact Metal–Organic Framework Single Crystals by Scanning Nuclear Microprobe
Source: J Am Chem Soc. 2021 Nov 2;143(44):18626–34. doi: 10.1021/jacs.1c08550 (PMC8587607; doi:10.1021/jacs.1c08550)
Supplement: Supplementary file 1 — ja1c08550_si_001.pdf [file ja1c08550_si_001.pdf]

Electronic Supporting information

Elemental depth profiling of intact metal-organic framework single crystals by scanning nuclear microprobe

Brian D. McCarthy,*[,a] Timofey Liseev,[a] Mauricio A. Sortica,[b] Valentina Paneta,[c] Wanja Gschwind,[a] Gyula Nagy,[c] Sascha Ott,*[,a] and Daniel Primetzhofer*[,b,c]

[a]Department of Chemistry – Ångström Laboratory, Uppsala University, Box 523, 751 20 Uppsala, Sweden. E-mail: brian.mccarthy@kemi.uu.se; sascha.ott@kemi.uu.se

[b]Tandem Laboratory, Uppsala University, Box 529, 751 20 Uppsala, Sweden.

[c]Department of Physics and Astronomy, Uppsala University, Box 516, 751 20 Uppsala, Sweden. E-mail: daniel.primetzhofer@physics.uu.se

Section	Pages
General methods	2-8
MOF ^{1}H-NMR analysis	10-12
Apparent diffusion coefficient estimation	13
References	13

General Methods

Instrumentation

X-ray diffraction (XRD) patterns were collected using a Siemens D5000 Kristalloflex equipped with a Gobel mirror (Bruker) and a parallel-plate collimator (0.40°) using a monochromatic Cu K_α radiation source (45 kV, 40 mA) for Cu Kα, (λ = 1.5406 Å) at a step size of 0.02º.

Scanning electron microscopy was performed on Carl Zeiss 1530 and 1550 SEM with InLens detector using 4.6 kV acceleration voltage.

^{1}HNMR analysis was performed with JEOL Resonance 400 MHz spectrometer.

Elastic backscattering spectrometry (EBS) experiments were performed using the microbeam line of the 5 MV 15SDH-2 pelletron accelerator at The Tandem Laboratory at Uppsala University, with a 5 MeV He beam focused in a 1,8 µm x 1,6 µm spot. An optical microscope is attached to the system and allows convenient beam positioning. During the measurements, the beam was set to scan a region of the sample, with an approximated area of 20 x 20 µm, containing one specific MOF crystal and scattered He from the sample were detected at a scattering angle of 170° by a solid-state detector. On this setup, the energy resolved scattering yield is recorded in coincidence with the X and Y coordinates of the beam, which permits creation of a 2-dimensional map of the chemical composition from 3-dimensional data on position and energy. Vice-versa, energy spectra can be extracted for later defined regions of interests.

Materials

3-iodobenzoic acid (Aldrich, 98%), 3-iodo-4-(methoxycarbonyl)benzoic acid (Sigma Aldrich, 90%), hafnium(IV) oxychloride hydrate (Sigma Aldrich, 99.99%), hydrofluoric acid (Sigma Aldrich, aqueous 48%), dimethylformamide (Merck, Supelco®), methanol (Merck, LiChrosolv®), zirconium(IV) oxynitrate hydrate (Sigma Aldrich, 99%), zirconium chloride (Sigma Aldrich, 99%), ethanol (VWR, 96%), and formic acid (Merck, 98-100%) were used as received. Diethylformamide (DEF) (Aldrich, 99%) was purified before use by stirring in activated charcoal for 30 minutes, then filtered twice through paper filters, centrifuged to collect, and passed through a 20 µm syringe filter. Anhydrous dimethylformamide (VWR, ≥99.7% unstabilised HPLC grade) was degassed with argon and further dried using an Inert Atmosphere solvent purification system. Iodoterephthalic acid was synthesized according to a literature procedure[1] from dimethyl iodoterephthalate (Fluorochem, 98%) and purity checked by ^{1}HNMR (**Figure S1**).

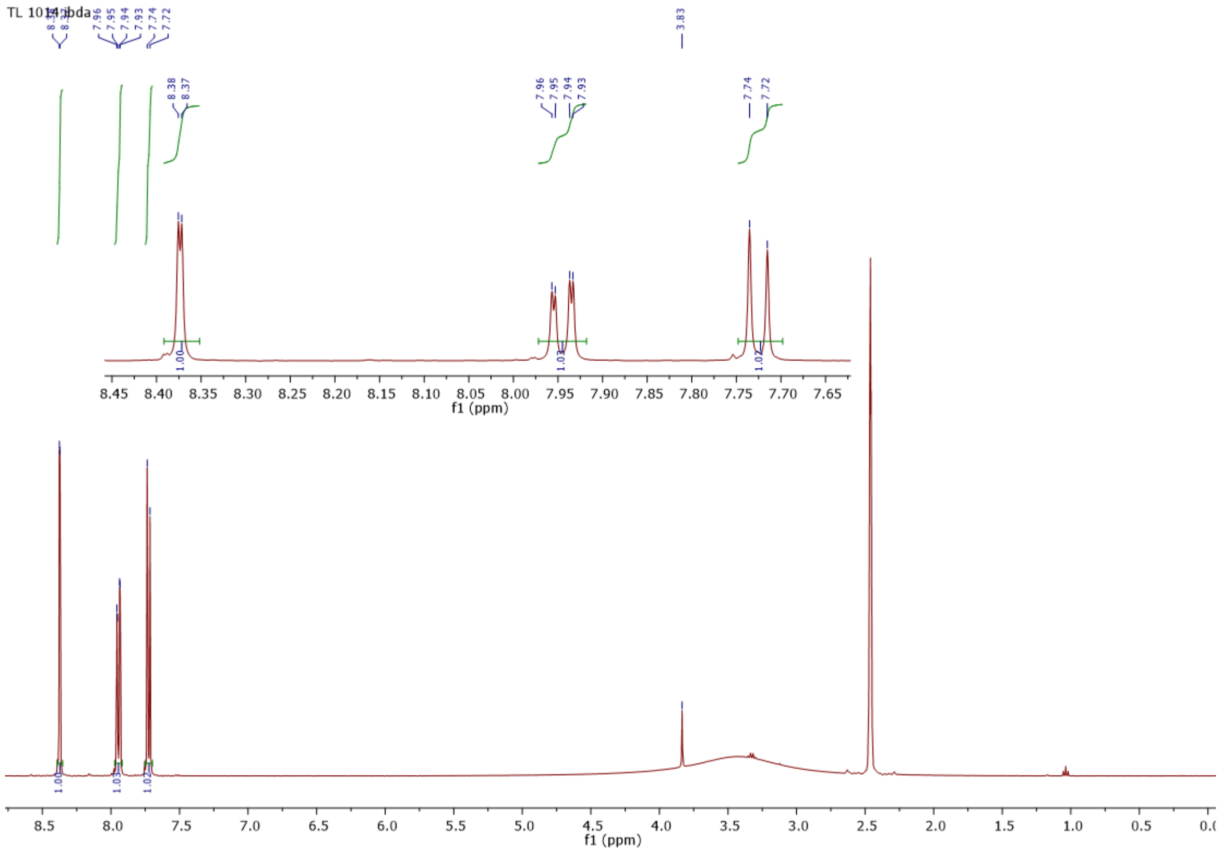

Figure S1. ^{1}HNMR spectrum of synthesized iodoterephthalic acid. (400 MHz, DMSO-d6) δ 8.37 ppm (1H, d, J = 1.5 Hz), 7.94 (1H, dd, J = 8.0, 1.5 Hz), 7.73 (1H, d, J = 8.0 Hz).

UiO-66@Si synthesis

Cut p-type (boron doped) single-side polished <100> Si slides (Siegert Wafer) were cleaned with piranha solution (a 3:1 mixture of concentrated H_2SO_4 and 30% aqueous H_2O_2) at 80 °C for 30 minutes, thoroughly rinsed with water, and blow-dried. A MOF precursor solution was prepared by mixing diethyl formamide solutions of $ZrOCl_2{\cdot}8H_2O$ (37 mM) and terephthalic acid (31 mM) with formic acid in a 1:1:1 ratio by volume. The Si slides were then submerged in the precursor solution (at a 45° tilt, polished side facing down to minimize bulk precipitation) and incubated in a preheated oven at 135 °C for 2 days. The obtained UiO-66@Si slides were washed and soaked at room temperature in DMF for 3 days and exchanged in DCM for 1 day. The resulting crystals were approximately 10-15 µm thick, 10-20 µm wide and highly oriented on the Si surface as demonstrated by SEM micrographs as well as the XRD pattern which shows almost exclusively the <111> orientation (**Figure S2**).

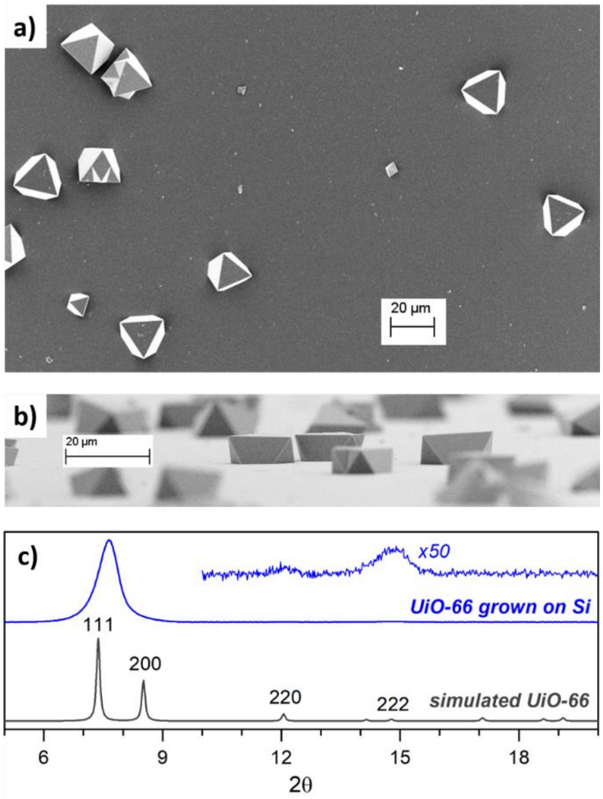

Figure S2. SEM images of surface-grown UiO-66@Si crystals in a) top view and b) side view, and c) PXRD patterns of as-synthesized pristine oriented UiO-66@Si compared to PXRD pattern of randomly oriented UiO-66 crystals simulated with Mercury[2] using a published UiO-66 structure[3] (structure file RUBTAK03 in the Cambridge Crystallographic Data Centre online database[4]).

Hf-doped UiO-66@Si synthesis

For the preparation of Hf doped UiO-66 large crystals, cut Si slides of ca. 0.7 x 2.2 cm were cleaned with piranha solution (3:1 v/v mixture of 30% aqueous H_2O_2 and fuming H_2SO_4) for 30 min at 80 °C. A MOF precursor solution was prepared by dissolving $ZrOCl_2·8H_2O$ (41.7 mg, 0.13 mmol) and $HfOCl_2·8H_2O$ (14.7 mg, 0.06 mmol) in diethylformamide (5 mL) and adding to it a solution of terephthalic acid (25.7 mg, 0.15 mmol) in diethylformamide (5 mL). The Si slides were rinsed with water, blow-dried, placed in a MOF vial (Supelco, 22 mL screw top vial, 20 mm screw cap with hole for PTFE/silicone septa, very loosely closed only) at a 45° tilt, polished side facing down, and submerged in the precursor solution. After 2 days of incubation at 135 °C the 30%Hf-UiO-66@Si slides were washed and soaked in DMF for 3 days and DCM for 1 day, exchanging for fresh solvent every day, before being dried under vacuum prior to ion beam analysis. The XRD pattern (**Figure S3**) matches well UiO-66 of strong 111 preferred orientation.

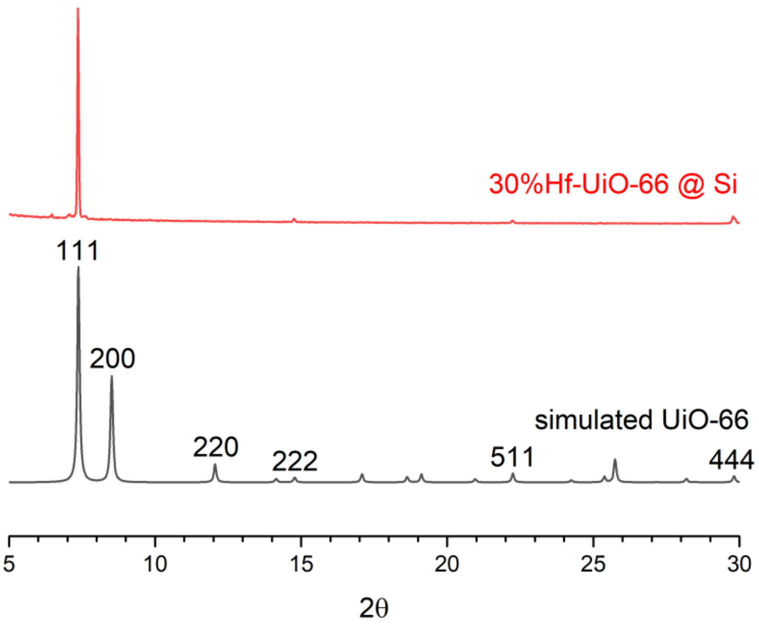

Figure S3. PXRD patterns of as-synthesized pristine oriented Hf-doped UiO-66@Si compared to PXRD pattern of randomly oriented UiO-66 crystals simulated with Mercury[2] using a published UiO-66 structure[3] (structure file RUBTAK03 in the Cambridge Crystallographic Data Centre online database[4]).

Post synthetic exchange of iodo-marked molecules into UiO-66@Si

For PSE, pre-evacuated UiO-66@Si slides were separately incubated with a 250 mM methanolic solution of iodo-labeled molecules (**Scheme S1**) 3-iodobenzoic acid (**iba**), iodoterephthalic acid (**ita**, Scheme 1) (synthesized by a reported method[1])), or 3-iodo-4-(methoxycarbonyl)benzoic acid (**mono-ester**) at 50 °C for 24 hrs, yielding UiO-66-**iba**, UiO-66-**ita**, and UiO-66-**mono-ester**, respectively. After the PSE process, the MOF@Si slides were washed in ethanol over 3 days, exchanging the solvent at least 6 times, followed by drying *in vacuo* before analysis.

Scheme S1. Iodine-labelled exchange molecules utilized for post-synthetic exchange.

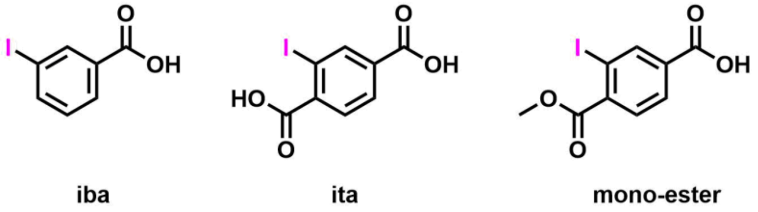

Table S1. Post-synthetic exchange conditions into UiO-66@Si crystals.

exchanging molecule	concentration	T	PSE duration	pre-PSE evacuation	iodine distribution
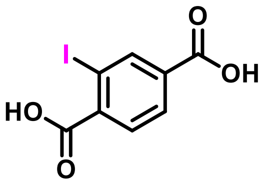	40 mM	25°C	24 hrs	-	*no iodine*
	100 mM	50°C	48 hrs	-	*no iodine*
	250 mM	50°C	24 hrs	24 hrs	iodine shell
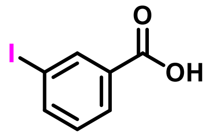	250 mM	50°C	24 hrs	24 hrs	iodine uniformly distributed

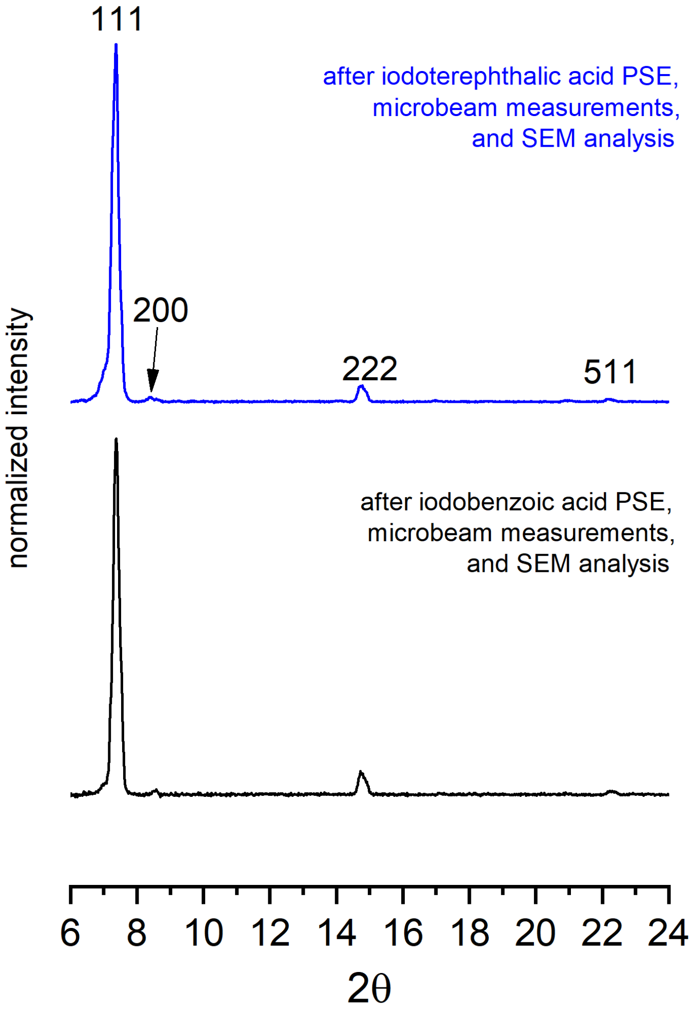

Figure S4. XRD patterns of UiO-66-**ita** (top) and UiO-66-**iba** (bottom), showing retention of crystal structure after PSE.

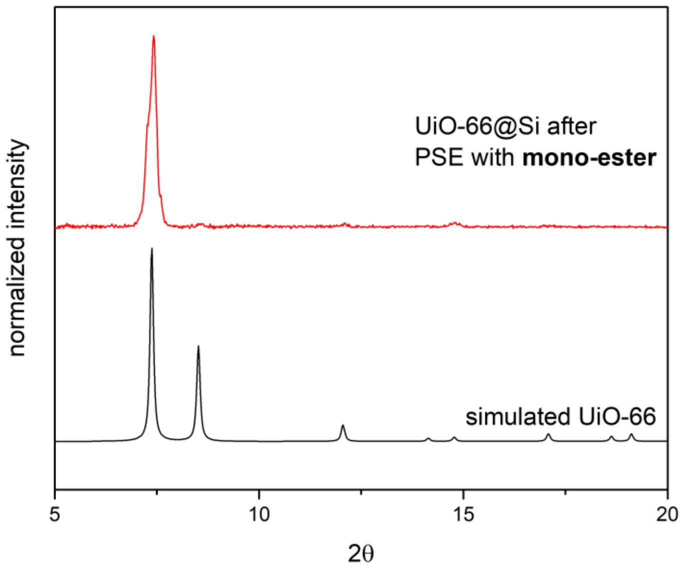

Figure S5. XRD pattern of UiO-66-**mono-ester**, showing retention of crystal structure after PSE.

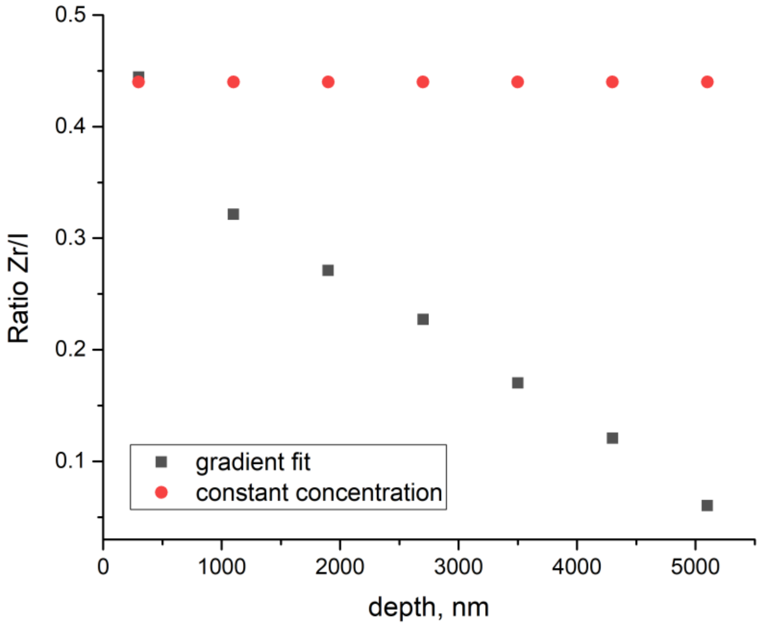

Figure S6. Ratio of Zr to I concentration as a function of depth for the two fits displayed in **Figure 5b** for UiO-66-**iba**.

Synthesis of UiO-67-bpy@Si

Silicon slides were prepared as before and placed polished-side down at a tilt inside 22 mL glass vials. A precursor solution was prepared as inspired by Long *et al*. for the growth of UiO-67-bpy single crystals:[5] 2,2′- bipyridine-5,5′-dicarboxylic acid (H_2bpydca, 46.3 mg, 0.2 mmol), benzoic acid (1.85 g, 15.2 mmol), and 15 mL anhydrous DMF were sonicated inside a dried 20 mL vial for several minutes. ZrCl4 (87.4 mg, 0.4 mmol) was added, the solution sonicated for a further minute before 24 µL water was added, and then 5 mL of the mixture was added to the silicon-slide containing 22 mL vial. Caps with septa were attached and the vials placed on a sand bath inside a 120 °C preheated oven. After 5 days the vials were removed, the

solution decanted, and the slides washed three times with anhydrous DMF, once with THF, and dried under vacuum prior to analysis. Note that due to the water sensitivity of UiO-67-bpy[6] we minimized the amount of time the samples were exposed to air. Analysis of the resulting slides revealed reflections characteristic of UiO-67 (**Figure S4**), and SEM imaging revealed a mixture of aggregated crystals and isolated single crystals presenting the 111 face (**Figure S5**).

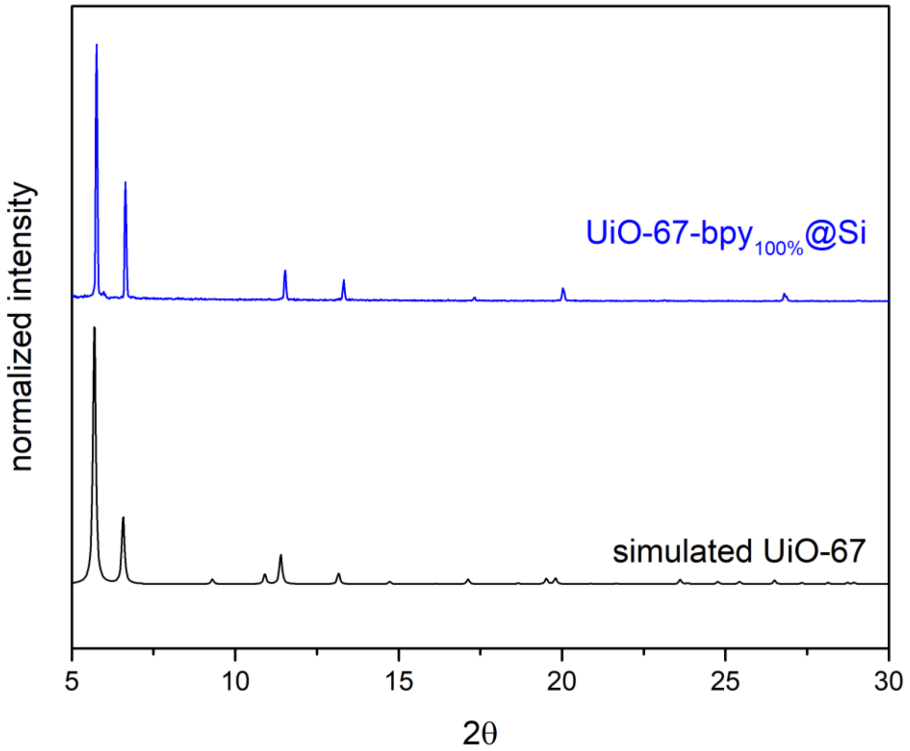

Figure S7. PXRD pattern of as-synthesized UiO-67-bpy@Si compared to the simulated pattern for UiO-67[7] (structure file WIZMAV01 in the Cambridge Crystallographic Data Centre online database).

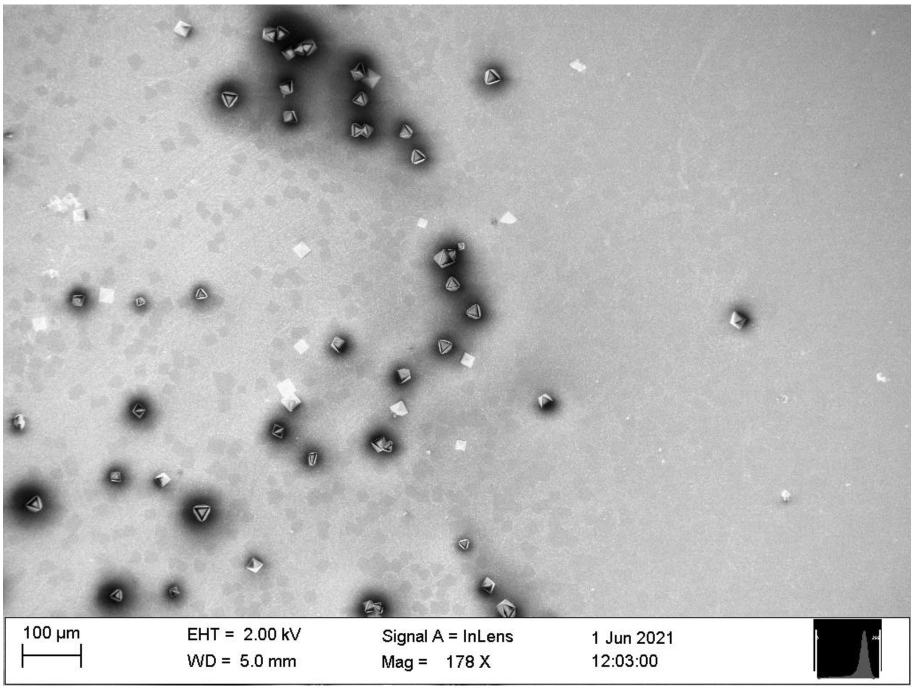

Figure S8. SEM image of UiO-67-bpy@Si.

Pt metalation of UiO-67-bpy@Si

The protocol of Øien was adapted.[8] Two UiO-67-bpy@Si samples which had been pre-evacuated were placed crystal-side up on the bottom of separate 20 mL glass vials. A 10 mL anhydrous DMF solution of K_2PtCl_4 (14.7 mg, 0.04 mmol) was prepared, and 5 mL of this solution was added to each 20 mL vial containing a UiO-67-bpy@Si sample. The vials were capped and placed in a dry block heater preheated to 100 °C for ca. 24 hours. The solutions were then decanted, and the samples heated in fresh DMF for one hour. The samples were transferred to clean 20 mL vials and washed six times with isopropyl alcohol over two days before being dried under vacuum. XRD (**Figure S9**) confirmed retention of the UiO-67 structure, though with decreased crystallinity we attribute to atmospheric moisture.

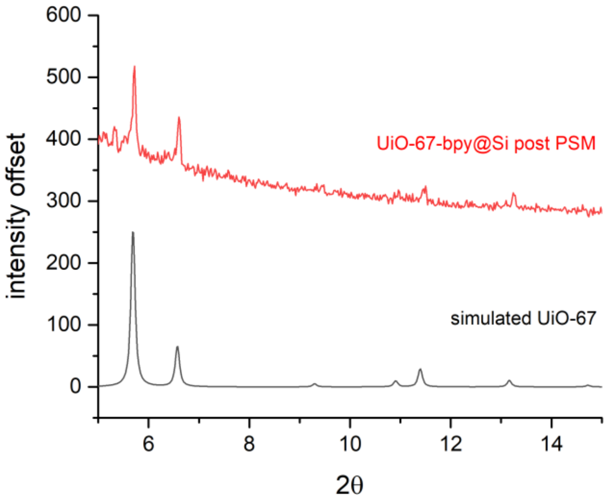

Figure S9. PXRD patterns of UiO-67-bpy@Si after PSM with K_2PtCl_4.

MOF ^{1}HNMR analysis

Bulk MOF preparation

Bulk UiO-66 MOF was synthesized by the same procedure used for the growth of UiO-66@Si.[9] After a 24 hrs drying under vacuum, two 10 mg portions of the bulk UiO-66 powder were subjected to PSE with **iba** and **ita** under the same conditions used for making UiO-66-**iba**@Si and UiO-66-**ita**@Si (250 mM of the exchanging molecule, 24 hrs, 50°C, followed by 3 days of washing in ethanol on a shaker, exchanging the solvent 6 times).

MOF digestion

Bulk samples of UiO-66, UiO-66-**iba** and UiO-66-**ita** were digested prior to NMR measurements. 5-10 mg of the MOF was suspended in 0.6 mL of dmso-d6 and 10 µL of 48% HF was added. The suspension was sonicated until no trace of the solid was observed.

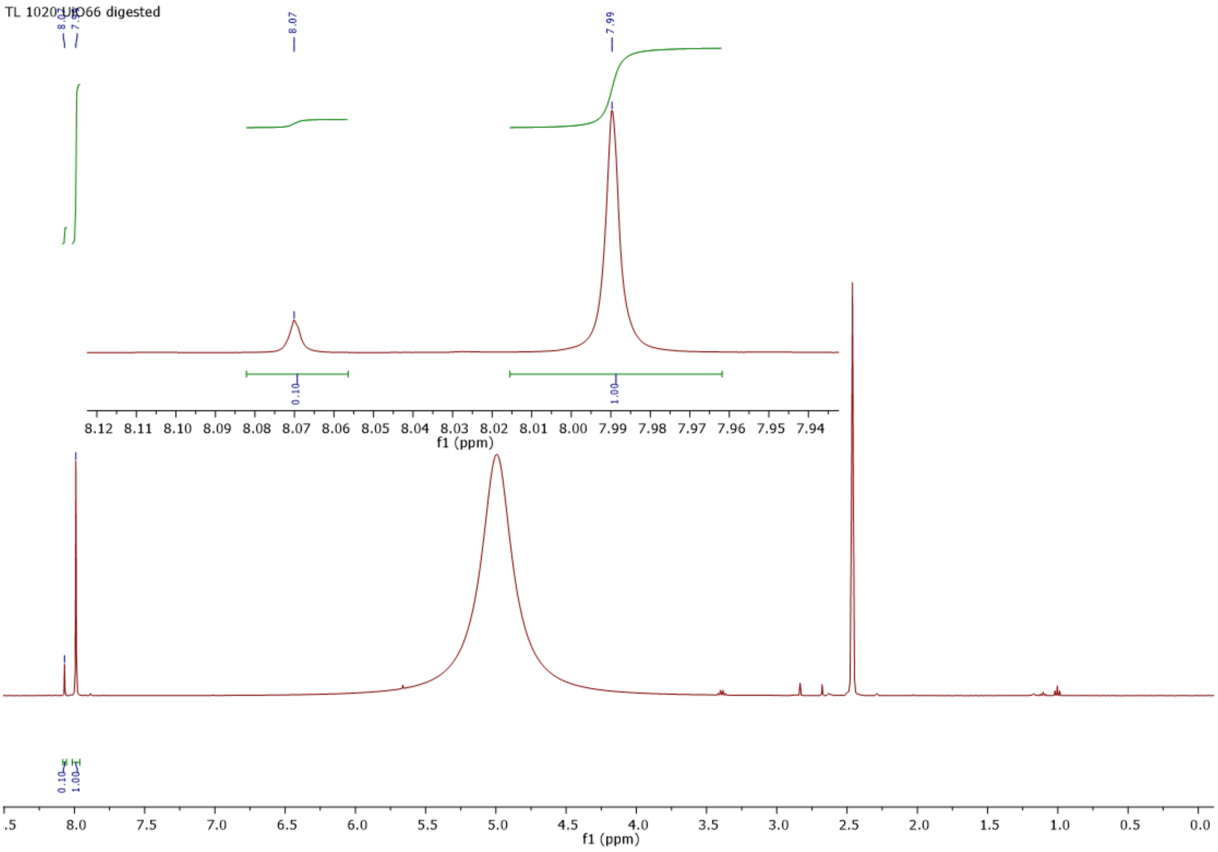

Figure S10. ^{1}HNMR spectrum of digested bulk UiO-66 MOF. The signal at δ 7.99 ppm corresponds to terephthalic acid (integral 1H), the signal at δ 8.07 ppm corresponds to formic acid (integral 0.1H).

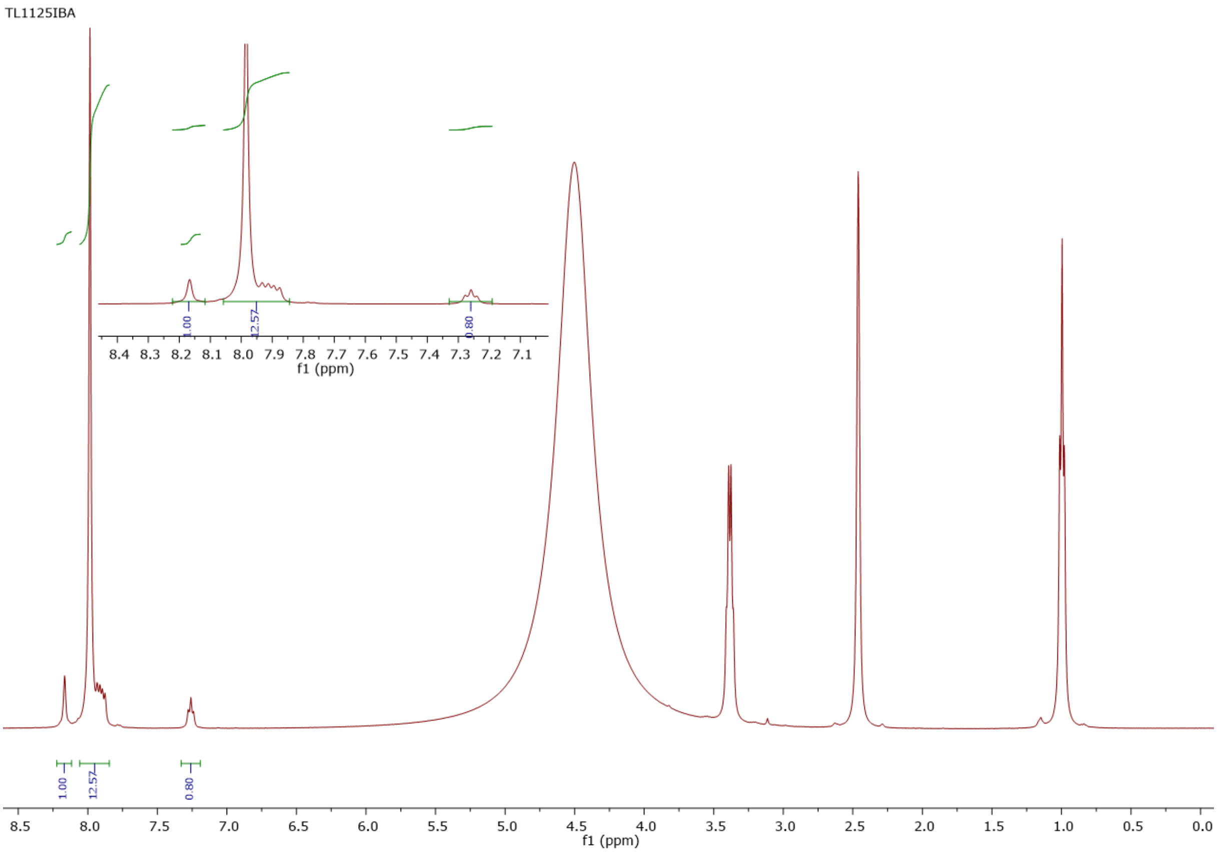

Figure S11. ^{1}H-NMR spectrum of digested bulk UiO-66-**iba** MOF. The singlet signal at δ 7.98 ppm corresponds to terephthalic acid. Iodobenzoic acid is present as four signals: a singlet at 8.17 ppm (1H), a triplet at 7.26 ppm (1H), and two doublets at 7.93-7.88 ppm (2H total). The latter signals (corresponding to two protons in ortho- and para-positions) intersect with the peak of the terephthalic acid. In order to quantify the amount of terephthalic acid, the entire multiplet at 7.98-7.88 ppm was integrated (12.6H) and the 2H intensity of the protons pertaining to **iba** was subtracted. The resulting intensity of the δ 7.98 ppm resonance is 10.6H.

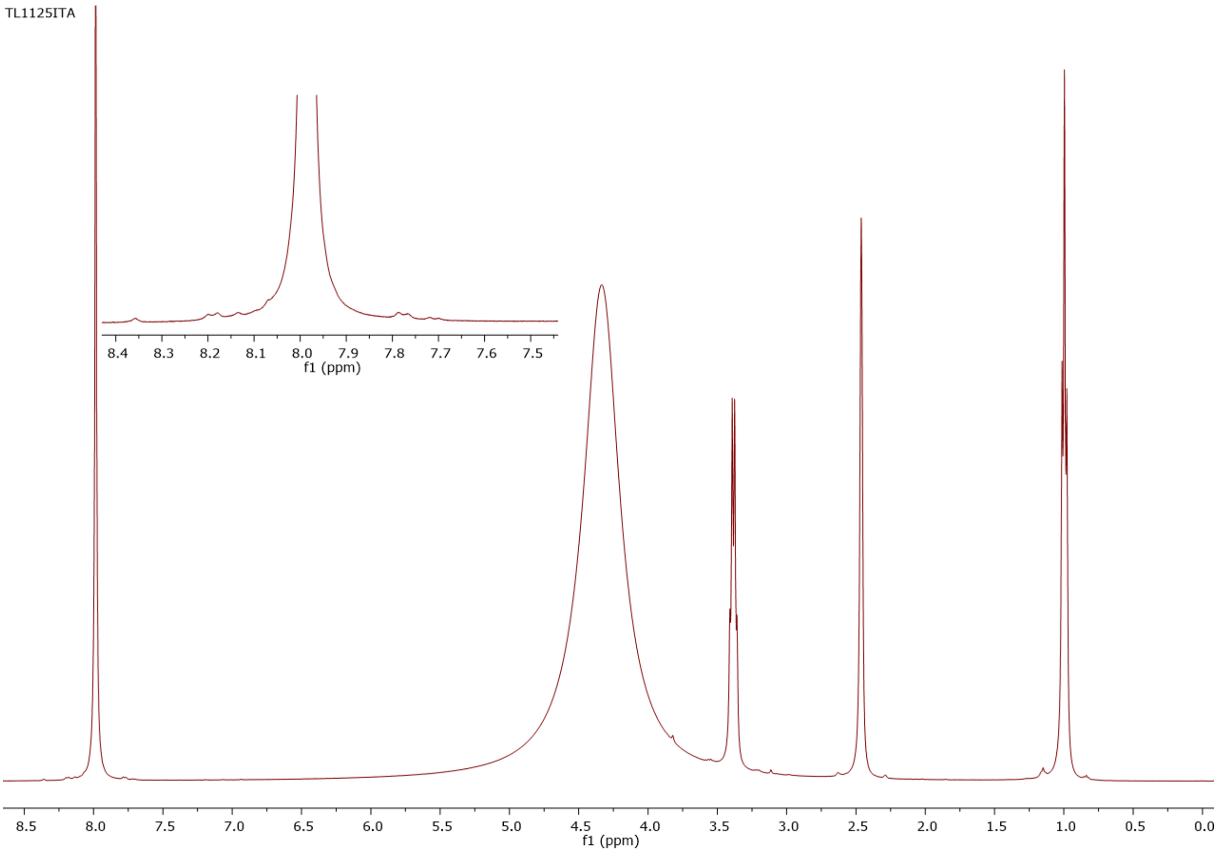

Figure S12. ^{1}H-NMR spectrum of digested bulk UiO-66-**ita** MOF.

Apparent diffusion coefficient estimation

For an order of magnitude estimation of the diffusion coefficient of **ita** within a MOF crystal, the following expression derived from the dimensional analysis of diffusion kinetics as described by J. Crank was used:[15]

$$T = const = \frac{Dt}{L^2},$$

where *T* is a dimensionless time parameter, *D* is the diffusion coefficient, *t* is time duration and *L* is distance. Applying the Buckingham π theorem, one can assume *T* = 1 in order to obtain a rough estimation of *D*:

$$D \approx \frac{L^2}{t}.$$

Setting *L* = 0,2 µm (the experimental **ita** shell thickness) and t = 24 hrs = 86400 s, the resulting diffusion coefficient can be estimated at ~10^{-19} m^2/s.

References

1 A. Kuttler, S. Durganala, A. Fratini, A. B. Morgan and V. Benin, *Heteroat. Chem.*, 2013, **24**, 361–371.

2 C. F. Macrae, I. Sovago, S. J. Cottrell, P. T. A. Galek, P. McCabe, E. Pidcock, M. Platings, G. P. Shields, J. S. Stevens, M. Towler and P. A. Wood, *J. Appl. Crystallogr.*, 2020, **53**, 226–235.

3 S. Øien, D. Wragg, H. Reinsch, S. Svelle, S. Bordiga, C. Lamberti and K. P. Lillerud, *Cryst. Growth Des.*, 2014, **14**, 5370–5372.

4 CCDC Access Structures, https://www.ccdc.cam.ac.uk/structures/, (accessed 11 December 2020).

5 M. I. Gonzalez, E. D. Bloch, J. A. Mason, S. J. Teat and J. R. Long, *Inorg. Chem.*, 2015, **54**, 2995–3005.

6 J. B. Decoste, G. W. Peterson, H. Jasuja, T. G. Glover, Y. G. Huang and K. S. Walton, *J. Mater. Chem. A*, 2013, **1**, 5642–5650.

7 S. Øien, D. Wragg, H. Reinsch, S. Svelle, S. Bordiga, C. Lamberti and K. P. Lillerud, *Cryst. Growth Des.*, 2014, **14**, 5370–5372.

8 S. Øien, G. Agostini, S. Svelle, E. Borfecchia, K. A. Lomachenko, L. Mino, E. Gallo, S. Bordiga, U. Olsbye, K. P. Lillerud and C. Lamberti, *Chem. Mater.*, 2015, **27**, 1042–1056.

9 C. A. Trickett, K. J. Gagnon, S. Lee, F. Gándara, H.-B. Bürgi and O. M. Yaghi, *Angew. Chemie Int. Ed.*, 2015, **54**, 11162–11167.

10 M. Taddei, *Coord. Chem. Rev.*, 2017, **343**, 1–24.

11 M. Taddei, R. J. Wakeham, A. Koutsianos, E. Andreoli and A. R. Barron, *Angew. Chemie Int. Ed.*, 2018, **57**, 11706–11710.

12 G. C. Shearer, S. Chavan, S. Bordiga, S. Svelle, U. Olsbye and K. P. Lillerud, *Chem. Mater.*, 2016, **28**, 3749–3761.

13 G. C. Shearer, J. G. Vitillo, S. Bordiga, S. Svelle, U. Olsbye and K. P. Lillerud, *Chem. Mater.*, 2016, **28**, 7190–7193.

14 M. J. Cliffe, W. Wan, X. Zou, P. A. Chater, A. K. Kleppe, M. G. Tucker, H. Wilhelm, N. P. Funnell, F. X. Coudert and A. L. Goodwin, *Nat. Commun.*, 2014, **5**, 1–8.

15 J. (Brunel U. U. Crank, *The Mathematics of Diffusion*, Clarendon Press Oxford, Second edi., 1975.
